# Genome Analyses of an Aggressive and Invasive Lineage of the Irish Potato Famine Pathogen

**DOI:** 10.1371/journal.ppat.1002940

**Published:** 2012-10-04

**Authors:** David E. L. Cooke, Liliana M. Cano, Sylvain Raffaele, Ruairidh A. Bain, Louise R. Cooke, Graham J. Etherington, Kenneth L. Deahl, Rhys A. Farrer, Eleanor M. Gilroy, Erica M. Goss, Niklaus J. Grünwald, Ingo Hein, Daniel MacLean, James W. McNicol, Eva Randall, Ricardo F. Oliva, Mathieu A. Pel, David S. Shaw, Julie N. Squires, Moray C. Taylor, Vivianne G. A. A. Vleeshouwers, Paul R. J. Birch, Alison K. Lees, Sophien Kamoun

**Affiliations:** 1 The James Hutton Institute, Invergowrie, Dundee, United Kingdom; 2 The Sainsbury Laboratory, Norwich Research Park, Norwich, United Kingdom; 3 SAC, Edinburgh, United Kingdom; 4 Agri-Food and Biosciences Institute, Belfast, United Kingdom; 5 USDA-ARS/PSI-GIFVL, BARC-West, Beltsville, Maryland, United States of America; 6 Horticultural Crops Research Laboratory, USDA ARS, Corvallis, Oregon, United States of America; 7 Emerging Pathogens Institute & Department of Plant Pathology, University of Florida, Gainesville, Florida, United States of America; 8 Biomathematics and Statistics Scotland, The James Hutton Institute, Invergowrie, Dundee, United Kingdom; 9 Escuela Politecnica del Ejercito, Sangolquí, Ecuador; 10 Wageningen UR Plant Breeding, Wageningen, The Netherlands; 11 The Sarvari Research Trust, Henfaes Research Centre, Abergwyngregyn, Llanfairfechan, United Kingdom; 12 Food and Environment Research Agency, Sand Hutton, York, United Kingdom; 13 Division of Plant Sciences, College of Life Sciences, University of Dundee at The James Hutton Institute, Invergowrie, Dundee, United Kingdom; CSIRO, Australia

## Abstract

Pest and pathogen losses jeopardise global food security and ever since the 19^th^ century Irish famine, potato late blight has exemplified this threat. The causal oomycete pathogen, *Phytophthora infestans*, undergoes major population shifts in agricultural systems via the successive emergence and migration of asexual lineages. The phenotypic and genotypic bases of these selective sweeps are largely unknown but management strategies need to adapt to reflect the changing pathogen population. Here, we used molecular markers to document the emergence of a lineage, termed *13_A2*, in the European *P. infestans* population, and its rapid displacement of other lineages to exceed 75% of the pathogen population across Great Britain in less than three years. We show that isolates of the *13_A2* lineage are among the most aggressive on cultivated potatoes, outcompete other aggressive lineages in the field, and overcome previously effective forms of plant host resistance. Genome analyses of a *13_A2* isolate revealed extensive genetic and expression polymorphisms particularly in effector genes. Copy number variations, gene gains and losses, amino-acid replacements and changes in expression patterns of disease effector genes within the *13_A2* isolate likely contribute to enhanced virulence and aggressiveness to drive this population displacement. Importantly, *13_A2* isolates carry intact and *in planta* induced *Avrblb1*, *Avrblb2* and *Avrvnt1* effector genes that trigger resistance in potato lines carrying the corresponding *R* immune receptor genes *Rpi-blb1*, *Rpi-blb2*, and *Rpi-vnt1.1*. These findings point towards a strategy for deploying genetic resistance to mitigate the impact of the *13_A2* lineage and illustrate how pathogen population monitoring, combined with genome analysis, informs the management of devastating disease epidemics.

## Author Summary

We have documented a dramatic shift in the population of the potato late blight pathogen *Phytophthora infestans* in northwest Europe in which an invasive and aggressive lineage called *13_A2* has emerged and rapidly displaced other genotypes. The genome of a *13_A2* isolate revealed a high rate of sequence polymorphism and a remarkable level of variation in gene expression during infection, particularly of effector genes with putative roles in pathogenicity. Collectively, these polymorphisms, in combination with an extended biotrophic phase, may explain the aggressiveness of *13_A2* and its ability to cause disease on previously resistant potato cultivars. The genome analysis identified conserved effectors that are sensed by potato resistance genes. These findings provide options for the strategic deployment of host resistance with a positive impact on crop yield and food security. This work stresses the benefits of a crop disease management strategy incorporating knowledge of the geographical structure, evolutionary dynamics, genome sequence diversity and *in planta*-induced effector complement of pathogen lineages.

## Introduction

As the cause of potato late blight, *Phytophthora infestans* is one of the most destructive plant pathogens within this genus of fungus-like oomycetes and widely known as the Irish potato famine pathogen [1], [2]. *P. infestans* has migrated from Central or South America [3], [4], where it infects many native solanaceous hosts, and remains responsible for significant losses to key staple crops (potato, tomato and other solanaceous plants) worldwide [5], [6]. Potato late blight management relies on regular applications of a range of anti-oomycete ‘fungicides’. However, under optimal weather conditions the pathogen may complete several infection cycles a week on a susceptible host, with control failure leading to rapid epidemics and crop loss. Resistance breeding offers great potential but the durability of resistance conferred by *R* genes has been continually challenged by the evolution of new virulence traits within pathogen populations [7]. *P. infestans* is normally diploid with a heterothallic (i.e. outbreeding) mating system that requires co-infection of A1 and A2 mating types to form long-lived sexual oospores. A mixture of sexually compatible A1 and A2 mating types increases the opportunities for sexual reproduction, providing the pathogen with an evolutionary advantage via increased genetic diversity and oospores as a source of primary inoculum in the soil [8], [9]. In the absence of oospores, in temperate regions the pathogen can only survive as asexual clones in potato tubers (as seed, in discard piles or unharvested tubers). Mycelium from such infections generates sporangia that are carried by wind and rain-splash to a new host where they germinate directly or release multiple motile zoospores that infect, colonize and release new sporangia via host stomata. Many studies have demonstrated that, despite the theoretical advantages of sexual recombination [8], a succession of clonal lineages of *P. infestans* have dominated the population in many potato production regions [7], [10]. In Europe, the incursion of the A2 mating type occurred 135 years after the A1 type [11]. However until recently, the A2 type remained infrequent in most parts of Europe [10], [12], which limited the opportunities for sexual reproduction of the pathogen [10], [13], [14]. Conversely, in parts of Mexico and the Nordic regions of Europe, populations of *P. infestans* have more balanced A1:A2 mating type ratios and are genetically diverse, with sexually formed oospores that act as a source of primary inoculum [7], [15].

Effective management of potato late blight is aided by an understanding of the characteristics of the contemporary pathogen population. For example, the aggressive and metalaxyl resistant A2 mating type *US-8* lineage replaced the *US-1* lineage which resulted in significant potato crop losses across the USA from 1985–1995 [16]. Pathogen genetic diversity has been monitored using a range of genetic markers [17] of which simple sequence repeats (SSRs) have recently proved effective for defining multilocus genotypes (MLGs) [18]. Key adaptive traits such as the ability of sporangia or zoospores to infect and colonise host tissue (aggressiveness) combined with efficient dissemination and, in temperate regions, survival from season to season (fitness) determine the success of particular *P. infestans* MLGs. Lesion growth rate and the period from inoculation to sporulation (latent period) are important components of aggressiveness [19], [20]. Fitness, a measure of reproductive success [21], is best studied in the field over several disease cycles. In a polycyclic disease such as potato late blight, even minor differences in aggressiveness or fitness can have a significant effect on the relative success of an MLG in the population. Traits such as ability to overcome specific host resistance, fungicide resistance or altered response to environmental conditions [22] are also important determinants of evolutionary success in the pathogen population.

The sequenced genome of *P. infestans* strain T30-4 provides a ‘blueprint’ of the gene complement and genome architecture of this pathogen [23]. The assembly served as a reference sequence in this work. Recently, two additional isolates PIC99189 and 90128 were resequenced using 36 bp Illumina reads (10.4× and 17.1× coverage, respectively) [24]. These projects revealed that *P. infestans* possesses a ‘two-speed genome’ with gene dense and gene-sparse repeat-rich regions. Gene-sparse regions (GSRs) are enriched in genes that are induced *in planta* and genes showing presence/absence polymorphism, copy number variation (CNV) or high nonsynonymous over synonymous substitution rates [24]. Effectors and other pathogenicity factors [23] that reside in these GSRs have the potential to evolve rapidly [24], consistent with the pathogen's well-documented capacity to adapt to novel host resistance. These effectors include RXLRs, a class of host translocated proteins that carry an N-terminal signal peptide followed by an RXLR motif [23], [25]. All known effector genes with Avr (avirulence) activity are *in planta*-induced genes of the RXLR type [26]. The study of the RXLR repertoire in emerging *P. infestans* lineages provides insights into the molecular basis of the infection phenotype on plants carrying the cognate *R* genes.

In the present study, we investigated changes in the population of the late blight pathogen *P. infestans* in Great Britain (GB) and identified a major new lineage of *P. infestans* that first emerged in mainland Europe in 2004. We investigated the factors driving this population change, demonstrating that *13_A2* MLG was amongst the most aggressive and fit MLGs in laboratory and field studies and able to overcome an important, previously durable source of host resistance. We sequenced the genome of an isolate of the *13_A2* MLG and compared it to the reference genome strain T30-4. We identified genes unique to this MLG, signatures of positive selection and CNVs, in particular in the RXLR effector repertoire. We also studied patterns of gene expression during an infection time course and noted a remarkable extended biotrophic phase, with distinct sustained induction of genes including RXLR effectors in the *13_A2* MLG isolate compared to other reference isolates. Lastly, we evaluated the effectiveness of promising sources of *R* genes that recognise invariant *Avr* genes, demonstrating that they remain effective against a *13_A2* MLG isolate. Despite the differential expression of many RXLR effector genes, we present evidence of a common set of *in planta*-induced effectors which we consider ‘targets’ for durable late blight disease resistance breeding.

## Results/Discussion

### Rapid and dramatic change in the Great Britain *Phytophthora infestans* population

We collected and determined the simple sequence repeat (SSR)-based [18] multilocus genotypes (MLGs) of 4,654 *P. infestans* isolates from 1,100 late blight disease outbreaks in Great Britain (GB), sampled between 2003 and 2008 (Table S1 in Text S2, Figure S1 in Text S1) cross-referencing these to a sample of isolates (*n* = 537) collected in previous GB surveys from 1982–1998 [13], [27], [28]. These SSR markers yielded between 2 and 25 alleles per locus and proved an effective tool to discriminate isolates within the GB pathogen population (Figure S2 in Text S1, Table S2 in Text S2).

**Figure 1:**
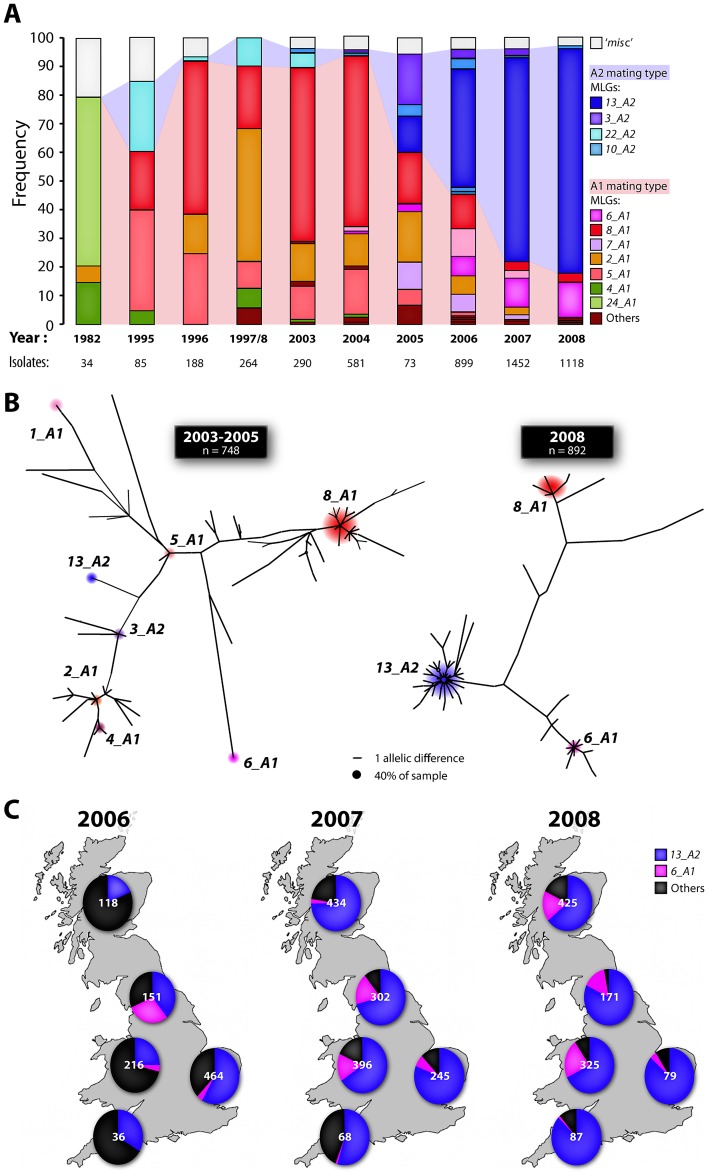
*P. infestans* population displacement in Great Britain by the *13_A2* genotype. (A) Frequency of multilocus genotypes (MLGs) over the course of 11 years from more than 4000 potato blight outbreaks. The number of isolates fingerprinted each year and dominant MLGs of each mating type are indicated. Isolates of MLGs that occurred at a very low frequency in a single year are grouped under the category termed *‘misc’*. The shading between the bars indicates the proportion of A1 and A2 mating type isolates. (B) Minimum Spanning Trees based on the alleles at 11 SSR loci indicating the relatedness of the main MLGs and decrease in population diversity between the periods 2003–5 and 2008. The numerous short branches from the *13_A2* MLG node reflect the high mutation rate in some SSR markers that results in intra-MLG diversity (*n* is the number of isolates from which the trees are derived). (C) Spatial pattern of spread two dominant MLGs across Great Britain (GB) from 2006–2008 (the numbers of isolates are indicated on each pie chart).

The *P. infestans* population was dominated by clonal lineages with fewer than seven MLGs comprising >82% of the isolates each year (Figure 1A, Table S3 in Text S2). The A2 mating type frequency increased and genetic diversity reduced markedly over the years 2005 to 2008 (Figure 1A and 1B, Figures S3, S4 in Text 1). A novel A2 mating type and metalaxyl resistant (Table S2A in Text S2) MLG, termed *13_A2*, was first recorded in seven British potato crops from July 2005 and went on to rapidly displace other MLGs across the region (Figure 1C). In 2006 MLG *13_A2* was prevalent in England from late May but not sampled in Scottish crops until late August (Figure S5 in Text S1) which is consistent with a progressive crop-to-crop dispersal across the region in 2006 (Figure 1C). Variation within the more variable SSR loci (particularly G11 and D13) has allowed discrimination of minor variants amongst the 2,295 isolates of *13_A2* MLG in this study (Figure 1B, Table S2B in Text S2). *P. infestans* MLG *13_A2* was first detected in isolates collected from The Netherlands and Germany in 2004, which is corroborated by other reports of A2 metalaxyl resistant isolates in continental Europe and suggests a north-westward migration to Great Britain (GB) (Table S4 in Text S2) [29]–[31]. The *‘misc’* category of SSR genotypes is a composite of all the novel and rarely sampled MLGs representing diversity that is consistent with sexual recombination [15]. However, in contrast to some other regions of Europe where almost every isolate is genetically distinct [15], this *‘misc’* category was recovered in GB disease outbreaks at a frequency of below 5% of the population from 2003 to 2008 (Figure 1A) indicating that the population remained largely clonal over this period (Figure 1B and Figure S4 in Text S1).

### *P. infestans* genotype *13_A2* is highly aggressive

We examined the selective forces behind the population displacement in extensive laboratory and field evaluations of the fitness of many isolates of *P. infestans*. Aggressiveness, ‘the quantity of disease induced by a pathogenic strain on a susceptible host’ [32], is a key component of pathogen fitness and was estimated by measuring lesion size and latent period (time elapsed from inoculation to spore production). Such adaptive traits contribute to the epidemiological success of this pathogen and closely correlate with spore production and infection frequency [19].

**Figure 2:**
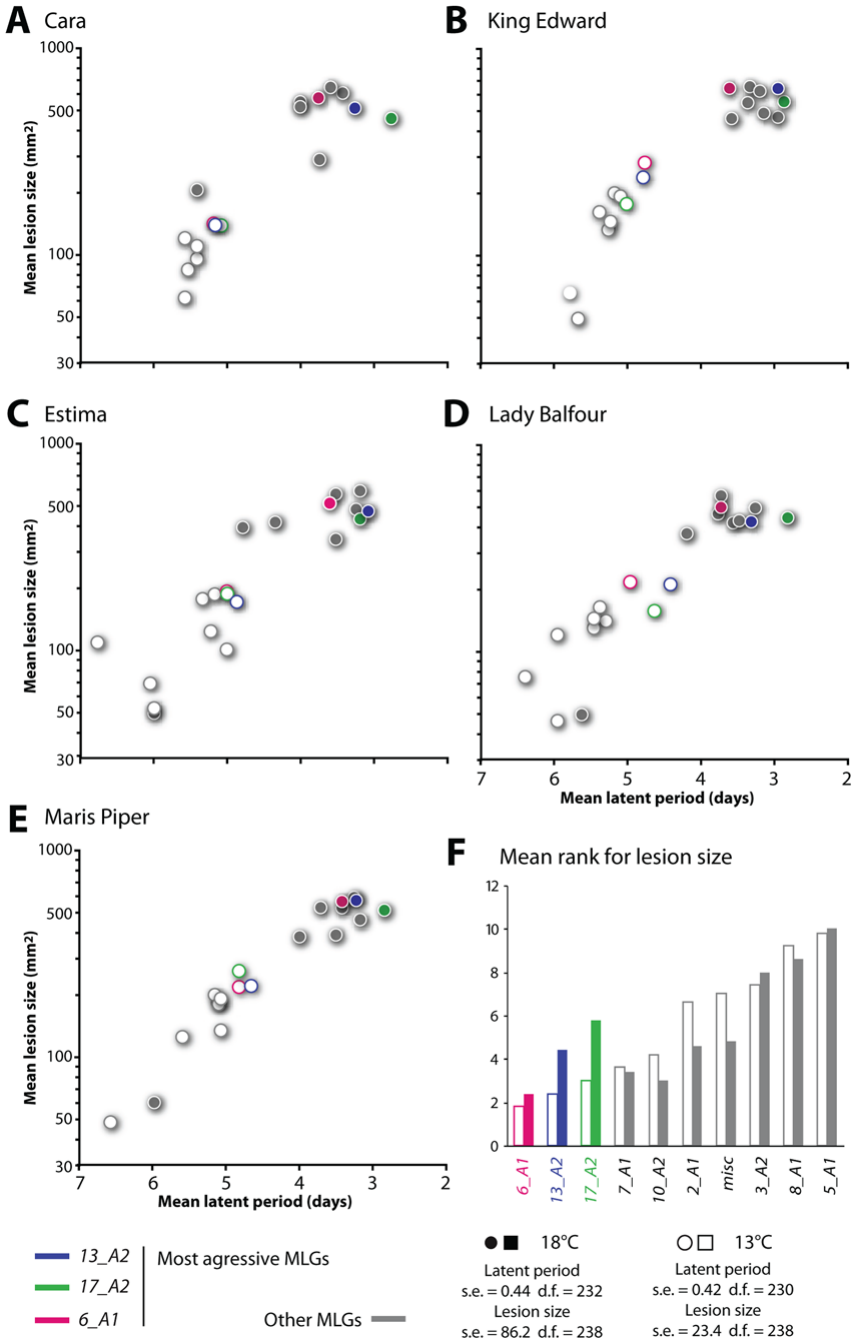
*13_A2* genotype is among the most aggressive *P. infestans* genotypes on potato. Aggressiveness of 26 *P. infestans* isolates grouped into 10 multilocus genotypes (MLGs) on leaves of five potato cultivars (A to E) estimated using mean latent period (x-axis) and mean lesion size at 6 days post inoculation (dpi) (y-axis). Measurements made at 13°C and 18°C are indicated with empty and filled symbols, respectively, and the three most aggressive MLGs colour-coded. (F) The sum of the ranked positions of each MLG according to lesion size at 13°C and 18°C indicates that *6_A1* and *13_A2* isolates more often had the largest lesions (particularly at 13°C). The standard errors (s.e.) and degrees of freedom (d.f.) for cultivar by MLG comparisons of latent period and lesion size at 13°C and 18°C (charts A to E) are shown in the lower corner of the figure.

A detached leaflet laboratory screen of 26 *P. infestans* isolates on five contemporary potato cultivars varying in foliar late blight resistance (Tables S5 and S6 in Text S2) was conducted at 13°C and 18°C. The isolates comprised representatives of the 9 MLGs in the 2006 British survey and reference isolates from other years and other European countries. MLG *13_A2* isolates consistently ranked among the most aggressive, showing among the shortest latent periods and the largest lesions of the MLGs tested, on all potato cultivars (Figure 2, Figures S6, S7, S8 in Text S1). This effect was more pronounced at 13°C than at 18°C, suggesting that MLG *13_A2* is better adapted to cooler conditions. Consistent with its frequency in the population (Figure 1C), MLG *6_A1* was also shown to be aggressive in this test.

Measurements of the lesion size produced on two different potato cultivars by a *13_A2* MLG isolate (06_3928A) and by the reference genome strain T30-4 [23], showed that 06_3928A formed larger lesions, with a shorter latent period than T30-4 (Figure S9 in Text S1). Also, we observed marked differences in the pattern of induction of the *Cdc14* gene in these two isolates during the biotrophic phase of infection on potato. This marker gene is associated with sporulation [33], and was induced earlier and more strongly in the biotrophic phase of infection by 06_3928A than by T30-4 which is consistent with the shorter latent period in 06_3928A (Figure S10 in Text S1).

### *P. infestans* genotype *13_A2* out-competes other aggressive genotypes

**Figure 3:**
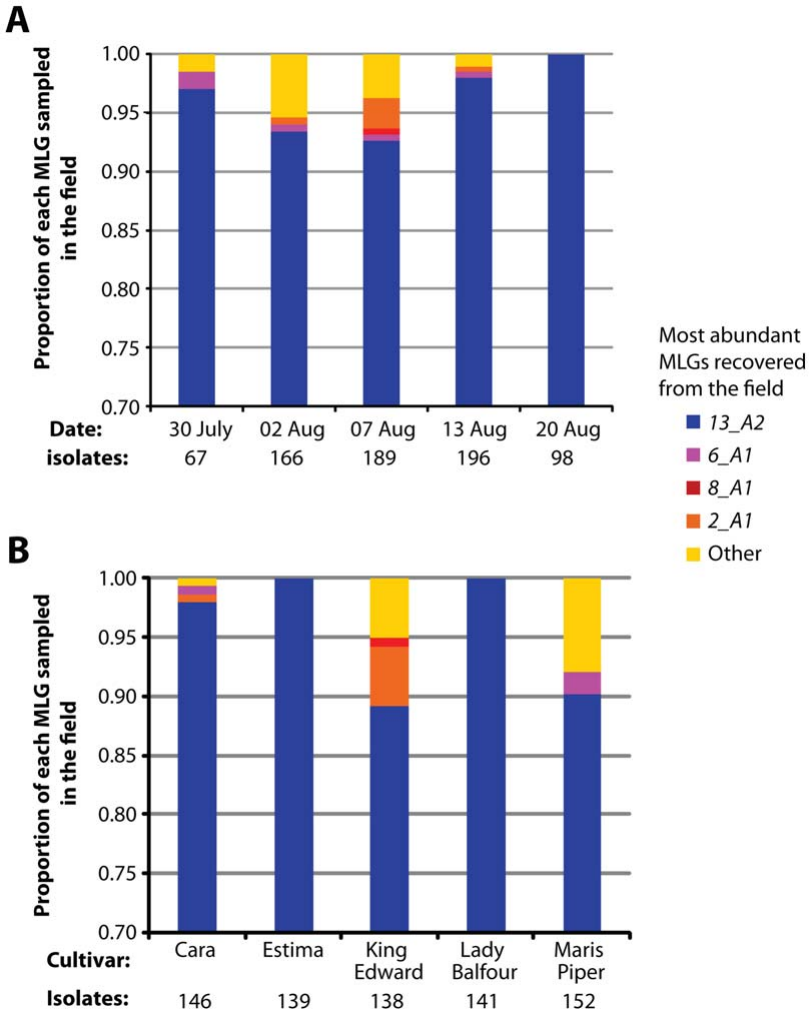
Field aggressiveness of five *P. infestans* genotypes estimated using mark-and-recapture methods during a controlled blight epidemic. The proportion of each multilocus genotypes (MLG) recovered amongst 716 foliar blight lesions over a 21 day epidemic initiated with 5 *P. infestans* isolates of known MLG presented by (A), sampling date and (B), Cultivar. Note; in each case the Y-axis scale is set from 0.7 to 1.0 to more clearly reveal the proportion of the non-*13_A2* MLGs. The isolates of ‘Other’ MLGs were of non-introduced clonal MLGs that migrated into the trial.

The above experiments demonstrate that, in a single disease cycle, *13_A2* isolates tend to be more aggressive than other MLGs under laboratory conditions. We went further to examine the ability of MLG *13_A2* to compete directly with other MLGs over many disease cycles in a field epidemic via a ‘mark and recapture’ experiment. The central potato plant of each of 20 field plots (five cultivars) was inoculated with a mixture of five isolates: *13_A2* (isolate 06_3928A) and representatives of four other contemporary MLGs, including *6_A1* (Table S5 in Text S2). Infected leaves from the ensuing epidemic were sampled over 21 days and 716 blight lesions were fingerprinted using direct SSR analysis. *13_A2* was the most prevalent MLG recovered, being responsible for the disease in 93–100% of the lesions sampled (Figure 3A). This high frequency was noted on all five cultivars which supported the field survey data showing a high recovery rate of *13_A2* MLG isolates from the ten most sampled cultivars (Figure 3B and Figure S11 in Text S1). In accordance with our results on the aggressiveness of *13_A2* at 13°C, the cool and wet conditions during the field trial (Figure S12 in Text S1) may have favoured the spread of MLG *13_A2*. Combined, these results provide strong evidence that isolates of *13_A2* MLG are more fit and aggressive than other MLGs on many host cultivars and under field and laboratory conditions, and are consistent with data on other *P. infestans* population displacements [34].

### *P. infestans* genotype *13_A2* overcomes resistance of the Stirling potato cultivar

**Figure 4:**
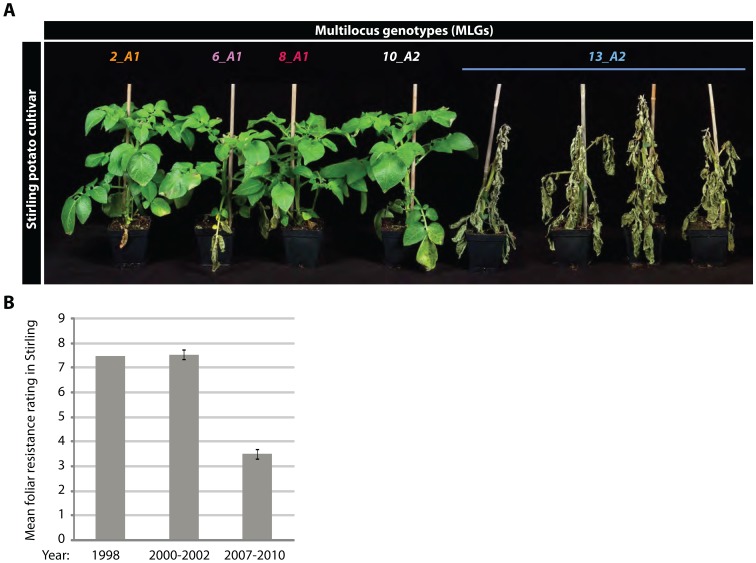
Breakdown of the blight resistance in Stirling potato cultivar by *13_A2* genotype. (A) Outcome of whole plant resistance tests of potato cultivar Stirling 7 days post inoculation with isolates of five multilocus genotypes (MLGs): 06_3888A (*2_A1*), 06_4100A (*6_A1*), 06_4256B (*8_A1*), 06_4440C (*10_A2*), 06_3928A, 06_4132E, 06_3964A and 06_3884B (*13_A2*). (B) Mean foliar blight resistance rating (on a 1–9 scale of increasing resistance) of potato cultivar Stirling in field trials conducted at two sites between 2000–2002 (n = 5) and between 2007–2010 (n = 4; after the introduction of MLG *13_A2*). The data are compared to the resistance rating of 7.5 reported in 1998. Error bar indicates s.e. = 0.3675 d.f. = 1.

In field trials since 2006, significant levels of disease were observed on some cultivars known to be partially resistant to foliar blight since the 1990s, such as Stirling [35] and Lady Balfour, a cultivar used in organic production. This was supported in subsequent whole-plant resistance screens which indicated a collapse of Stirling's resistance (Figure 4). We examined the ability of many isolates of *13_A2* MLG to overcome foliar late blight resistance on eleven potato *R* differential plants that contain immune receptor genes derived from the Mexican species *Solanum demissum*. All isolates of *13_A2* were able to cause disease on all the differential plants, except *R8* and *R9* (Table S5 in Text S2 and Figure S13 in Text S1). This indicates that, in addition to being particularly aggressive on susceptible potato cultivars, isolates of *13_A2* caused more disease on a broader spectrum of late blight resistant potato cultivars than isolates belonging to other *P. infestans* MLGs.

### Genome sequence of isolate 06_3928A of *P. infestans* genotype *13_A2*

In late blight resistant potato plants, hypersensitive cell death and resistance are triggered by recognition of specific pathogen RXLR effectors by matching R proteins [26]. Effectors are pathogen proteins delivered inside plant cells to promote host colonization, for instance by suppressing plant immunity [36]. RXLR proteins, encoded by ∼563 genes in the *P. infestans* T30-4 genome [23], are the main class of host translocated effectors. Some RXLR effectors are said to have an “avirulence” activity when acting as triggers of plant immunity. To determine the genetic features, in particular the effector gene repertoire, associated with the *13_A2* MLG phenotype, we generated ∼58-fold genome coverage Illumina paired-end reads of isolate 06_3928A (see details in Text S3). We processed the sequences first by aligning the reads to the reference genome of *P. infestans* strain T30-4 [23], and then by performing *de novo* assembly of unaligned reads. In total, 95.6% of the 06_3928A reads aligned to the T30-4 sequence (Table S7 in Text S2). We detected 18,106 coding sequences with an average breadth of coverage of 99.2% (Table S8 in Text S2). We optimized bioinformatic parameters for calling single nucleotide polymorphisms (SNPs) to reach 99.9% accuracy and 85.8% sensitivity (Figure S14 in Text S1). Using these parameters, we identified 22,433 SNPs in 5,879 coding sequences of 06_3928A (Tables S8 in Text S2 and Table S9). This is similar to the 20,637 and 21,370 SNPs reported for *P. infestans* isolates PIC99189 and 90128, respectively [24] (Table S8 in Text S2). Of the total SNPs discovered, 11,795 were unique to 06_3928A among the four examined strains, indicating a considerable degree of variation in the *13_A2* isolate (Table S9 and Figure S15 in Text S1).

### High dN/dS rates are frequent among RXLR effectors in *P. infestans* genotype *13_A2* isolate 06_3928A

**Figure 5:**
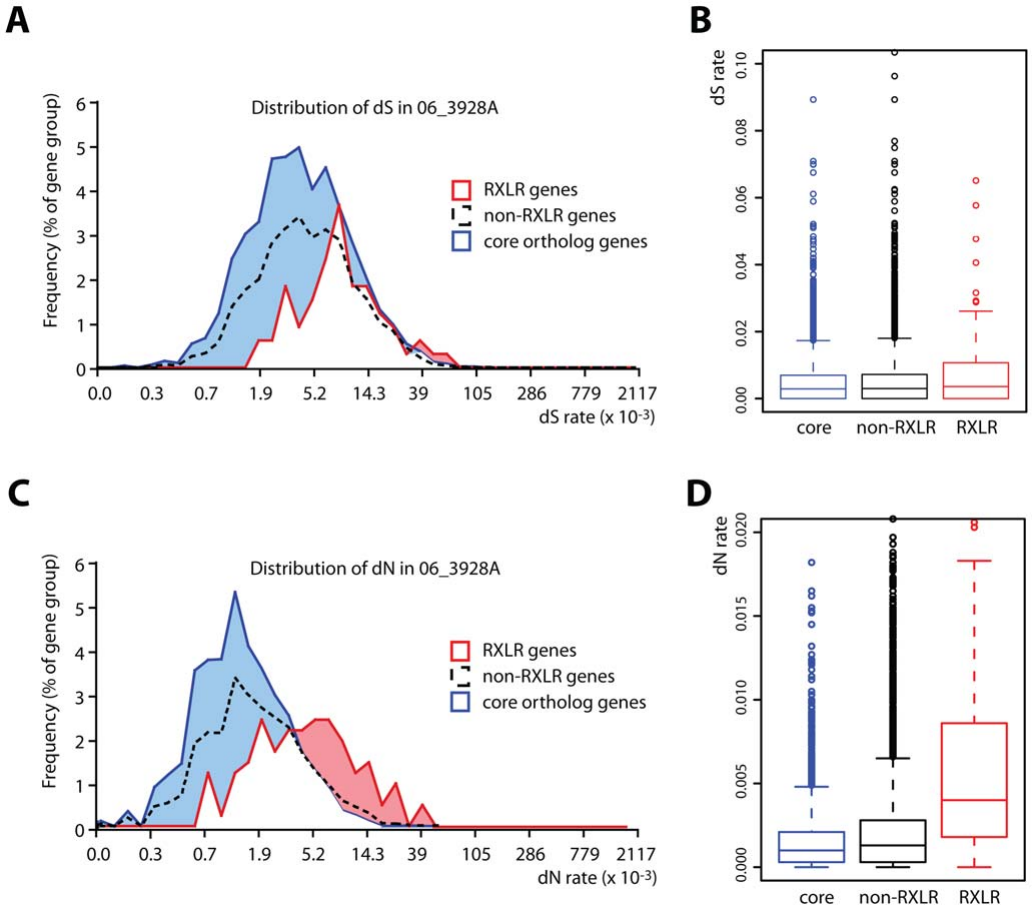
Distribution of polymorphism in genes of *P. infestans 13_A2* isolate 06_3928A. Y-axis indicates the frequency of synonymous substitutions (dS) in (A) and nonsynonymous substitutions (dN) in (C) given for RXLR genes, other (non-RXLR) genes and core ortholog genes [23]. Y-axis shows the rates of synonymous substitutions (dS) in (B) and nonsynonymous substitutions (dN) in (D) given for RXLR genes, other (non-RXLR) genes and core ortholog genes. Box and whisker plots in (B and D) show median, first and third quartile, and first values beyond 1.5 times the interquatile range.

**Figure 6:**
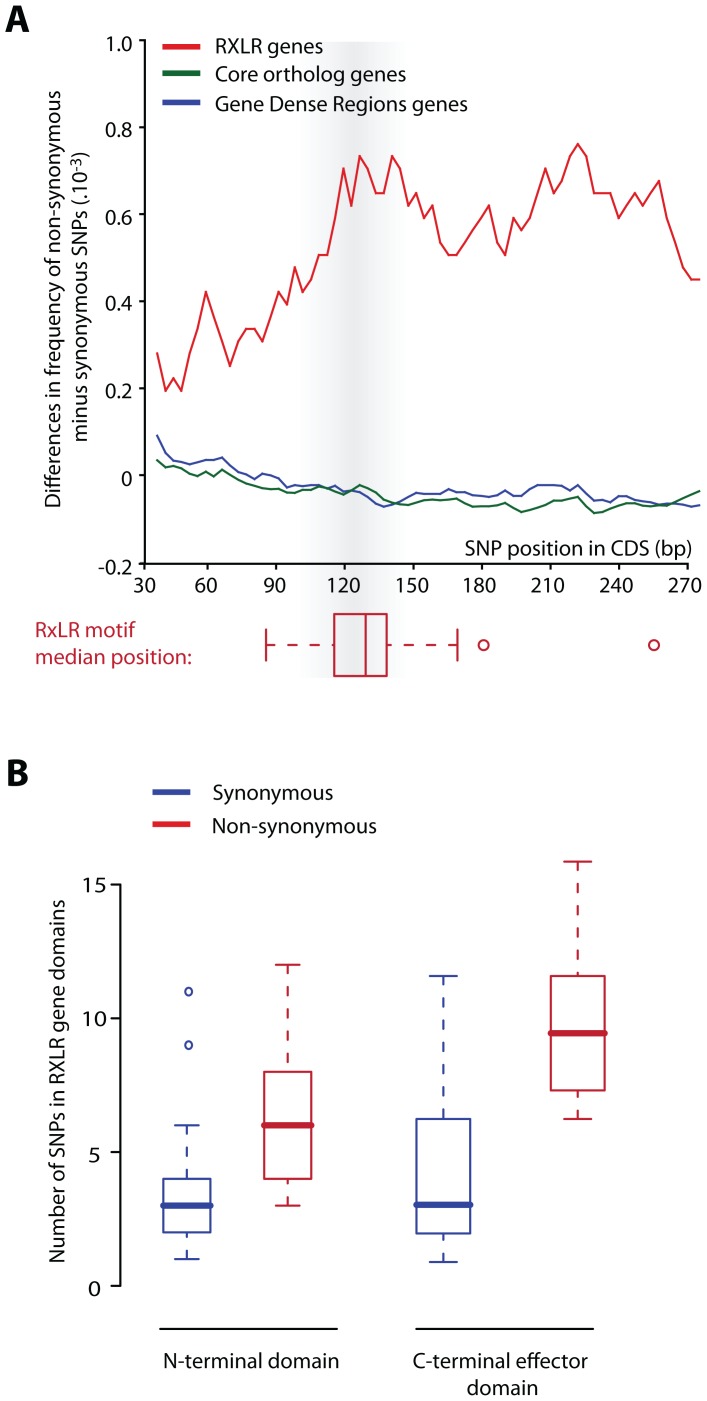
Distribution of single nucleotide polymorphisms (SNPs) along the coding sequence (CDS) of RXLR effector genes in *P. infestans 13_A2* isolate 06_3928A. (A) Summary of nonsynonymous and synonymous SNPs in CDSs (all genes, core orthologs and RXLRs) of 06_3928A compared to T30-4 reference genome strain. Core orthologs as genes showing orthologous sequences 1∶1∶1 in *P. infestans: P. ramorum: P. sojae* genomes respectively [23]. (B) Distribution of the number of synonymous and non-synonymous SNPs in the N-terminal and C-terminal domain of RXLR effector genes in 06_3928A isolate. Box and whisker plots show median, first and third quartile, and first values beyond 1.5 times the interquartile range.

To detect signatures of positive selection in the *13_A2* lineage, we calculated rates of synonymous (dS) and nonsynonymous (dN) substitutions for every gene (Table S10). Of the 22,523 coding sequence SNPs, 11,421 are nonsynonymous (51%) corresponding to an average dN/dS rate of 0.34. Secreted protein genes, particularly RXLR effector genes, show higher dN rates compared to other categories (Figure 5). Of the 405 SNPs detected in RXLR genes, 278 are non-synonymous (69%) corresponding to an average dN/dS rate of 0.53 (Table 1 and S11). RXLR effectors are modular proteins with N-termini involved in secretion and host-translocation while C-termini encode the effector biochemical activity [25], [37]. The C-terminal domains of RXLR effector genes are highly enriched in nonsynonymous substitutions as previously noted in other oomycete species (Figure 6) [38]. Several RXLR effector genes show high dN/dS ratios and multiple replacements in their C-terminal domain (Figure S16A–C in Text S1). In addition to RXLR effectors, other secreted proteins including a Kazal-like serine protease inhibitor show high dN/dS ratios (Figure S16D in Text S1). These amino acid polymorphisms could contribute to the enhanced aggressiveness and virulence phenotypes of this genotype.

**Table 1:** Summary of nonsynonymous and synonymous single nucleotide polymorphisms (SNPs) in coding genes (CDSs) of *P. infestans* 06_3928A compared to T30-4 reference genome strain.

SNP count*	All genes	Core orthologs	RXLRs
Total No. of SNPs in coding genes	22,433	11,612	405
Total No. of nonsynonymous SNPs in coding genes	11,421	5,439	278
Total No. of synonymous SNPs in coding genes	11,012	6,173	127
No. of genes with at least one SNP	5,879	2,754	118
Average dN/dS†	0.34	0.3	0.53

* count of SNPs causing loss of stop codons were omitted;

† dN/dS rates were calculated using Yang method reported in Yang and Nielsen [56].

Nonsynonymous and synonymous SNPs were calculated for all genes, core orthologs and RXLRs. Core orthologs as genes showing orthologous sequences 1∶1∶1 in *P. infestans: P. ramorum: P. sojae* genomes respectively [23].

### Copy number variations are frequent among RXLR effectors genes in *P. infestans* genotype *13_A2* isolate 06_3928A

To estimate copy number variation (CNV) in the resequenced genome of *P. infestans 13_A2* isolate 06_3928A relative to T30-4, we used average read depth per gene and GC content correction (see Text S3). We detected 367 CNV events in 06_3928A genes, of which there are 320 duplications and 47 deletions (Tables S12, S13). In agreement with other studies [23], [24] genes showing deletions and duplications occur more frequently in the plastic gene sparse regions of the 06_3928A genome (Figure S17 in Text S1). RXLR effector genes show higher rates of CNV compared to other gene categories (Figure S18 in Text S1 and Table S13). We identified two RXLR effectors with ∼4–5 additional gene copies in the isolate 06_3928A and this was validated with a realtime PCR assay in 17 of 18 other isolates of *13_A2* MLG. Another 18 *P. infestans* MLGs had lower copy numbers suggesting the higher copy number duplications are unique to *13_A2* MLG isolates (Figure S19 in Text S1). Remarkably, 21% (10 out of 47) of the genes that are deleted in 06_3928A encode RXLR effectors (Table S14 in Text S2). *13_A2* MLG isolates are able to infect potatoes carrying the *R1* gene (Figure S13 in Text S1) which is consistent with our finding of an ∼18 Kb deletion encompassing the region surrounding the *Avr1* RXLR effector gene in the 06_3928A isolate (Figure S20 in Text S1) [26], [39].

### Assembly of unmapped reads from *P. infestans* genotype *13_A2* isolate *06_3928A* reveals novel candidate RXLR effector genes

To identify sequences that are unique to 06_3928A, we performed *de novo* assembly of the unmapped Illumina reads and identified a total of 2.77 Mb contigs that did not align to *P. infestans* T30-4 sequences. *Ab initio* and homology based gene calling in these 06_3928A-specific contigs revealed 6 candidate RXLR effector genes absent in the T30-4 reference genome strain (Table S14 in Text S2). All 6 RXLR genes were subsequently confirmed by PCR on genomic DNA to be present in the 06_3928A isolate and absent in T30-4 (see Text S3, Table S15 in Text S2). Among these, a highly divergent homolog of *Avr2* evades recognition by the *R2* resistance gene and explains the virulence of 06_3928A on *R2* potatoes (Tables S14, S15 in Text S2 and Figure S13 in Text S1) [26], [40]. Interestingly, the PCR testing also showed that the six novel RXLR genes in the 06_3928A isolate of *13_A2* MLG are present in various combinations in other multilocus genotypes (MLGs) sampled from Great Britain. This illustrates the heterogeneity of the RXLR effector repertoire that can occur within the wider *P. infestans* population. These findings point to a series of genetic polymorphisms that collectively contribute to the aggressiveness and virulence phenotype of the *13_A2* MLG.

### Gain and loss of gene induction in RXLR effectors of *P. infestans 13_A2* isolate 06_3928A

**Figure 7:**
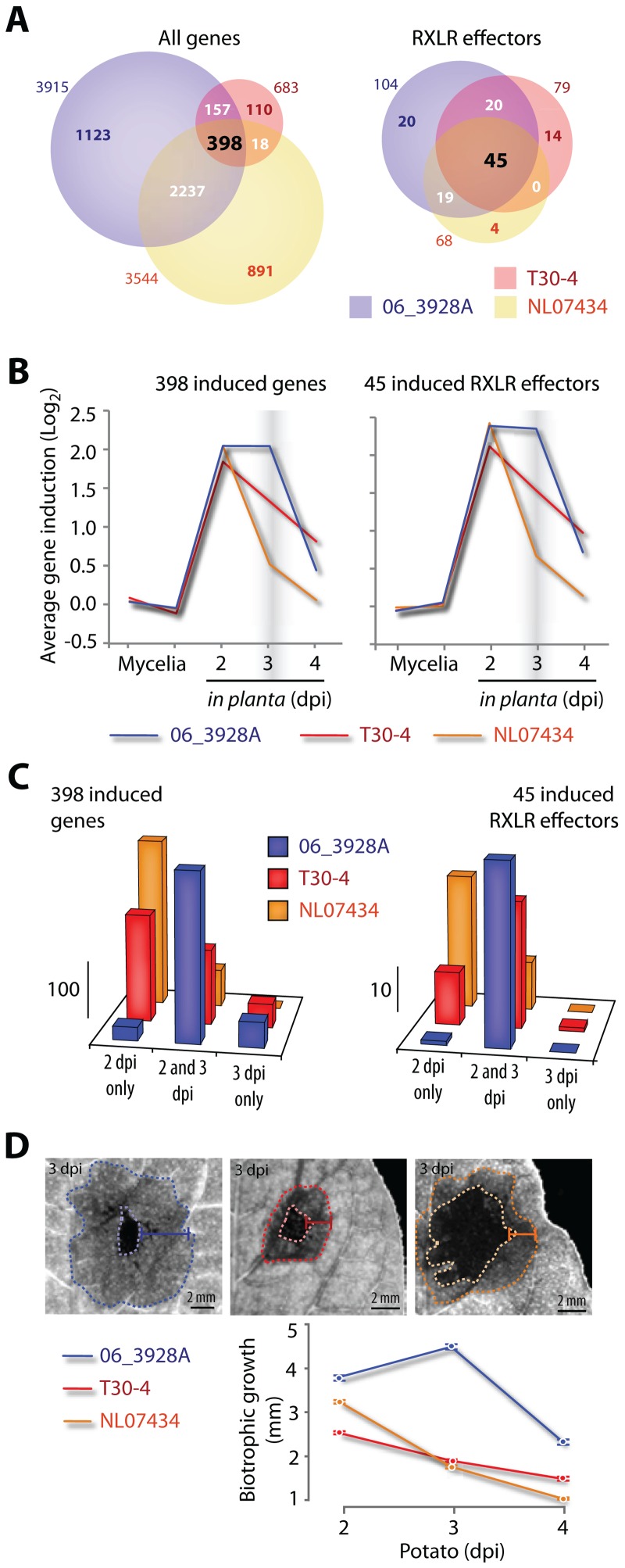
Gene expression polymorphisms correlate with extended biotrophy in *P. infestans 13_A2* isolate 06_3928A. (A) Number of genes (left) and RXLR effector genes (right) that are induced during potato infection in *P. infestans* T30-4, 06_3928A and NL07434 strains. Only a small subset of genes is consistently induced in the three strains. (B) Average gene expression pattern during potato infection for all genes (left) and RXLR effector genes (right) induced in all three *P. infestans* strains analyzed. A consistent divergence is observed at 3 days post inoculation (dpi) when gene induction is maintained in the 06_3928A isolate only. (C) Number of all (left) and RXLR effector genes (right) induced at various time points during potato infection in each of the three *P. infestans* strains. Compared to other strains, 06_3928A shows the highest number of genes that are induced both at 2 and 3 dpi. (D) Variation in the size of the biotrophic area (infected living host tissue) in lesions induced by three *P. infestans* strains during potato infection. Error bars are s.e.m. over 28 measurements at 2, 3 and 4 dpi. Representative pictures illustrate the sizes of the necrotrophic (infected dead host tissue, centre of the lesion) and biotrophic (periphery of the lesion, lighter grey ring) growth (mm) with the respective *P. infestans* strain in color (blue for 06_3928A, red for T30-4 and orange for NL07434 strain) from lesions at 3 dpi.

The phenotype of the *13_A2* MLG may not only result from changes in gene coding sequences as documented above, but also from changes in gene expression. An infection time course was performed by hybridizing NimbleGen microarrays with cDNA from potato leaves harvested at 2–4 days post inoculation (dpi) with *P. infestans* 06_3928A, the T30-4 reference genome strain, and a third strain, NL07434, collected in 2007 in The Netherlands (see Text S3). We observed frequent expression polymorphisms between the three strains with 1,123 genes specifically induced in 06_3928A, compared with 110 in T30-4 and 891 in NL07434 (Figure 7A, Table S16). Remarkably, only 398 out of 4,934 genes were induced in all three strains indicating distinct isolate-specific sets of genes induced during potato infection (Figure 7A). *P. infestans* effector genes are sharply induced during the biotrophic phase of infection, when the pathogen associates closely with living plant cells [23], [26]. We identified 104 RXLR effector genes that are induced during biotrophy in 06_3928A compared to only 79 and 68 in T30-4 and NL07434, respectively (Figure 7A, Table S11). Of these 104 RXLR genes, expression of 20 was specifically detected in the 06_3928A isolate but not in the other two (Figure 7A, Figure S21 in Text S1). In contrast, 18 RXLR effector genes are not induced in 06_3928A but are induced in at least one of the other two isolates (Figure 7A). One of these genes, *Avr4* is recognized by the *R4* resistance gene [26], [41]. The lack of induction of *Avr4* in 06_3928A (Figure S21 in Text S1) is consistent with the virulence of *13_A2* isolates on plants containing *R4* (Figure S13 in Text S1). The updated repertoire of RXLR effectors and their expression profiles presented in this study provides additional data for systems biology approaches to understanding the role of effectors in plant-microbe interactions [42].

### *P. infestans 13_A2* isolate 06_3928A shows patterns of sustained gene induction and extended biotrophic growth during potato infection

We noted a distinct temporal pattern of *in planta* gene induction in 06_3928A. Most up-regulated genes in this isolate showed sustained induction over 2 and 3 dpi in contrast to T30-4 and NL07434, in which transcript abundance generally declines at 3 dpi (Figure 7B–C, Table S16) coinciding with the onset of host tissue necrosis [23]. These findings prompted us to determine the extent to which gene induction patterns and disease progression correlate in 06_3928A and these other isolates. Microscopic observations of lesions caused by 06_3928A revealed significantly larger biotrophic zones during infection (Figure 7D). The genes showing a sustained induction period in 06_3928A include putative virulence factors such as RXLR effectors, cell wall hydrolases, proteases and protease inhibitors (Table S16). The extended biotrophic phase of 06_3928A during host plant colonization, combined with expression of a range of effectors and other secreted virulence determinants, likely contribute to the enhanced aggressiveness (Figure 2) and field fitness of MLG *13_A2* isolates. However, additional work is required to determine exactly which genes contribute to MLG *13_A2* aggressiveness and fitness.

### Exploiting the RXLR effector repertoire to manage *P infestans 13_A2* epidemics

**Figure 8:**
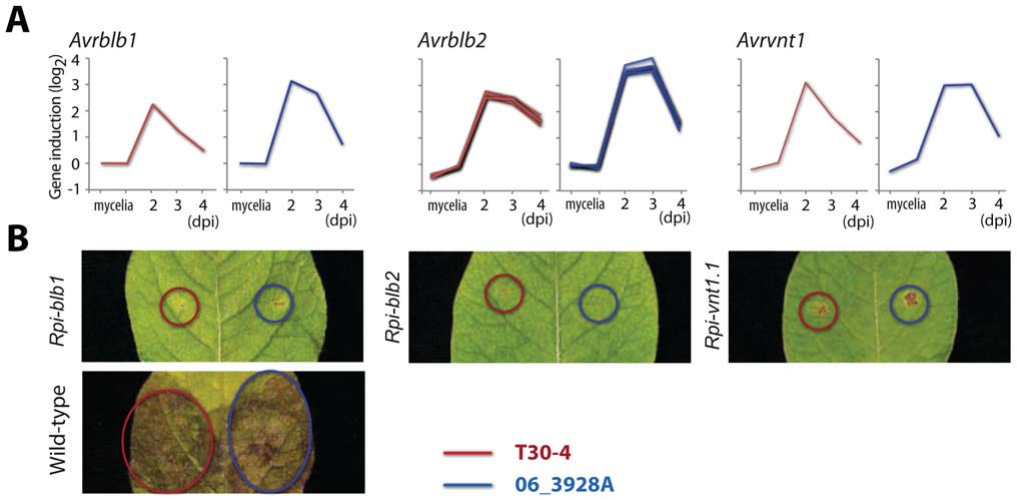
Invariant avirulence genes in *P. infestans* 06_3928A identify efficient plant resistance genes. (A) Expression patterns of three avirulence genes in *P. infestans* T30-4 (red) and 06_3928A strains (blue) during potato infection. In addition to sequence conservation, these genes also show conserved gene induction patterns in 06_3928A. (B) The corresponding potato resistance genes confer resistance to *P. infestans* T30-4 and 06_3928A strains.

The genome analyses of MLG *13_A2* offers opportunities to identify useful forms of host resistance. The 45 “core” RXLR effectors showing *in planta* gene induction during biotrophy in all 3 examined strains include 5 known avirulence effector genes (Figure 7A). Whilst homologs of *Avr2* [40] and *Avr3a* [43] in the 06_3928A isolate contain sequence polymorphisms and are known to evade recognition in plants carrying the corresponding *R2* and *R3a* genes (Figure S13 in Text S1), *Avrblb1* [44], *Avrblb2* [45] and *Avrvnt1* [46] occur as intact coding sequences that are induced during infection (Figure 8A). These three *Avr* effectors are therefore predicted to be recognized by their cognate immunoreceptors. To determine whether *13_A2* MLG can infect plants carrying the *Rpi-blb1*, *Rpi-blb2* and *Rpi-vnt1.1* resistance genes, we used isolate 06_3928A to inoculate stable transformant potato cv. Desiree lines expressing, independently, each of the three *R* genes. In each case, 06_3928A was unable to infect the *R* potatoes and triggered a typical hypersensitive response (Figure 8B) indicating that the three *R* genes are effective against this *13_A2* MLG isolate. Such sources of resistance will thus be an effective component of any integrated management system against late blight caused by genotype *13_A2*.

### Conclusions

We report the emergence of an aggressive clonal lineage of *P. infestans*, multilocus genotype (MLG) *13_A2*, and its rapid displacement of other genotypes within the Great Britain (GB) population. MLG *13_A2* has overcome previously durable disease resistances in potato, such as in cultivar Stirling and is resistant to phenylamide fungicides. Late blight caused by this lineage has thus proved challenging to manage and its migration to other potato growing regions of the world poses a threat to sustainable crop production. Therefore, there is a need, when developing a strategy for deploying disease resistance, to identify and respond rapidly to dramatic changes, and new epidemics caused by emerging genotypes within the pathogen population. Genome analyses of the *13_A2* isolate 06_3928A revealed a high rate of sequence variation and a remarkable pattern of extended biotrophic growth, which may explain *13_A2's* aggressiveness and ability to cause disease on previously resistant potato cultivars. The genome analysis proved valuable in identifying RXLR effectors sensed by potentially durable potato resistance genes. This stresses the benefits of a crop disease management strategy incorporating knowledge of the geographical structure and evolutionary dynamics of pathogen lineages combined with data on their genome sequence diversity (and *in planta* induced effector gene complement). Such data, when linked to the host *R* gene repertoire [47], offers options for strategic deployment of host resistance with a positive impact on crop yield and food security.

## Materials and Methods

### Pathogen surveillance and isolate characterisation

*P. infestans* isolates were obtained from more than 1,100 outbreaks of potato late blight across Great Britain (GB) from 2003 to 2008. The locations of 672 outbreaks sampled in 2006 to 2008 and further details on sampling and pathogen characterisation are available (Figure S1 in Text S1 and Text S3). The mating type of each of 4,654 isolates collected in this study was tested by pairing with known tester isolates on Rye A agar plates. After an initial screen of the new A2 mating type lineages using the RG57 [48] RFLP probe (Table S2A in Text S2), all isolates were genotyped using 11 SSR markers [18] in 3 multiplexed PCR assays using fluorescently labelled primers on an ABI 3730 capillary sequencer (Tables S2 and S3 in Text S2 and Text S3). The SSR data were used to define MLGs, explore the relatedness amongst the multilocus genotypes (MLGs) and to describe the population change. Due to the presence of three alleles in some isolates, we calculated clonal distance [49] using the infinite alleles mutation model, to quantify genetic distance between MLGs. This distance essentially counts the number of alleles that differ between individuals. Isolates with null alleles were included, but any isolates that were not genotyped at one or more loci were excluded. Distance among multilocus genotypes was calculated in GenoDive (Distributed by P. G. Meirmans at http://www.bentleydrummer.nl/software/software/Home.html). Minimum spanning networks were calculated by MINSPNET [50] and visualized using neato in the Graphviz package [51]. The numbers of isolates used to construct the trees were 748, 795, 1,072, and 892 for 2003–2005, 2006, 2007, and 2008, respectively (Figure 1B and Figure S4 in Text S1).

### Aggressiveness and fitness testing in the laboratory and field

Representative isolates from the main MLGs from Great Britain plus a selection of reference isolates from other countries were used to examine two components of aggressiveness [19] (lesion size and latent period) on five contemporary potato cultivars (Tables S5 and S6 in Text S2) as follows. For each cultivar, leaflets of a similar age and size were placed in clear plastic boxes (26 leaflets per box) lined with moist tissue paper. After chilling to stimulate zoospore release, a droplet of 30 µl of inoculum (approx 420 sporangia) of each of the 26 isolates was applied to the centre of each leaflet. A total of 60 boxes of leaves were inoculated and 30 placed in a randomised block design with six replicate blocks in each of two adjoining illuminated walk-in growth rooms set at a constant 13°C or 18°C with 16/8 hours of light and dark. The 1,560 leaflets were scored daily for first symptoms (i.e. infection period, IP), and sporulation (i.e. latent period, LP) and at six days post inoculation (dpi), lesion size was measured in two orientations at right angles to each other using electronic calipers connected to a laptop computer.

A randomised block field trial comprising four replicate 25 plant plots of the five potato cultivars used in the laboratory assay was established. In mid-July an equal mixture of sporangia of 5 isolates (different MLGs) were used to infect the lower leaves of the central plant in each plot. Once the disease had spread from the central plant, single lesions were sampled from the epidemic over the following three weeks and direct SSR fingerprinting of *P. infestans* from lesions pressed onto FTA cards (Whatman, UK) was used to determine the MLG. For additional details see Text S3.

### Whole-genome sequence analysis

Genome sequencing of *P. infestans 13_A2* isolate 06_3928A was performed in 2G GAs (Illumina Inc.) and alignments were obtained with Burrows-Wheeler Transform Alignment (BWA) software package v0.5.7 with a seed length (l) of 38 and a maximum of mismatches (M) allowed of 3 as parameters [52]. Unmapped reads of *P. infestans 13_A2* isolate 06_3928A were assembled using VELVET software package v1.0.18 [53] and mapped to the reference genome using NUCmer program from MUMmer software package v3.2 (see details in Text S3) [54].

A False Discovery Rate (FDR) analysis was used to determine the performance of single nucleotide polymorphism (SNP) calling in the 06_3928A genome (Figure S14 in Text S1 and Text S3). Single nucleotide polymorphisms (SNPs) were called using a 90% consensus among reads calling a SNP with a minimum of 10× coverage (Figure S16 in Text S1). Rates of synonymous substitution (dS), non-synonymous substitution (dN) and omega (dN/dS) were calculated using the yn00 program of PAML [55] by implementing the Yang and Nielson method [56] for every coding gene predicted in 06_3928A in comparison to the homologous gene in the reference genome strain T30-4 (Figure 5, Table S10). Differences in frequencies of nonsynonymous minus synonymous SNPs were counted per 15 bp-long windows and sliding by 3 bp steps. Frequencies were calculated as the number of SNPs per bp per gene and averaged over 20 consecutive windows (Figure 6A). The 20 windows adjacent to the RXLR motif were considered for each of the domains. Numbers of SNPs in RXLR gene domains were counted per 15 bp-long windows and sliding by 3 bp steps (Figure 6B). A total of 118 RXLRs, 3,077 core orthologs and 2,442 gene-dense regions (GDR) genes that contain at least 1 SNP were analyzed (Figure 6).

### Whole-genome expression profiling

The NimbleGen microarray data are available in GEO under accession number GSE14480 for *P. infestans* T30-4 [23] and GSE33240 for *P. infestans* 06_3928A and NL07434. Genes that are induced *in planta* were identified using a t-test (p value<0.05, >2 fold expression changes) and False Discovery Rate (FDR) analysis (q-value<0.05) [57] in samples from infected potato leaves relative to plate-grown in mycelia (see more details in Text S3).

## Supporting Information

Text S1 **Supplementary Figures S1–S21.**
(PDF)

Text S2 **Supplementary Tables S1–S8 and S14–S15.**
(DOC)

Text S3 **Supplementary Materials and Methods; (S3a) Pathogen sampling and genotyping: (S3b) Aggressiveness and virulence testing: (S3c) Pathogen whole-genome and expression analyses.**
(DOC)

Table S9 **List of single nucleotide polymorphisms (SNPs) detected in** ***P. infestans 13_A2*** **isolate 06_3928A genome.** This list includes details of SNPs calculated in coding sequences (CDSs) from 06_3928A genome. SNPs described as unique in 06_3928A are those SNPs that were not found in the isolates 90128 and PIC99189 [24]. A SNP was estimated when on 90% of the aligned bases encoded for that SNP with a minimum read depth of 10. This list excludes 90 SNPs causing loss of stop codons out of the 22,523 total number of SNPs detected in all coding sequences of 06_3928A isolate.
(XLS)

Table S10 **Polymorphism data associated to each gene of** ***P. infestans 13_A2*** **isolate 06_3928A genome.** The table provides the number of all single nucleotide polymorphisms (SNPs), the number of synonymous SNPs, the number of nonsynonymous SNPs, the rate of synonymous (dS), the rate of nonsynonymous (dN), and the dN/dS ratio estimated in each of the coding gene sequences (CDSs) from 06_3928A isolate.
(XLS)

Table S11 **List of genome features and expression profiles of RXLR effectors of** ***P. infestans 13_A2*** **isolate 06_3928A.** This list includes as features of RXLRs: 1) the presence of secretion signals [23], [58]; 2) whether they belong to the 1∶1∶1 *Phytophthora* spp. core orthologs (*P. infestans: P. sojae: P. ramorum*) [23]; 3) RXLR family; 4) gene environment based on intergenic distances [24]; 5) presence/absence polymorphism according to average breadth of coverage (e.g. 0% is considered as missing); 6) the predicted number of additional gene copies in the genome (e.g. 1 is equivalent to one additional gene copy); 7) number of single nucleotide polymorphisms (SNPs), 8) number of nonsynonymous SNPs; 9) number of synonymous SNPs; 10) omega (dN/dS); 11) nonsynonymous dN rates; 12) synonymous dS rates and 13) if there is gene induction in potato.
(XLS)

Table S12 **List of 320 genes of** ***P. infestans 13_A2*** **isolate 06_3928A showing duplications copy number variation CNV>1 (genes with at least one additional gene copy predicted in 06_3928A).** This list includes for each of the 320 coding genes: 1) annotations [23]; 2) the presence of secretion signals [23], [58]; 2) whether they belong to the 1∶1∶1 *Phytophthora* spp. core orthologs (*P. infestans: P. sojae: P. ramorum*) [23]; 3) the effector type; 4) RXLR family [23]; 5) gene environment based on intergenic distances [24]; 6) the predicted number of additional gene copies in the genome (e.g. 1 is equivalent to one additional gene copy).
(XLS)

Table S13 **List of 47 genes of** ***P. infestans 13_A2*** **isolate 06_3928A showing presence absence polymorphisms.** This list includes for each of the 47 coding genes: 1) annotations [23]; 2) the presence of secretion signals [23], [58]; 2) whether they belong to the 1∶1∶1 *Phytophthora* spp. core orthologs (*P. infestans: P. sojae: P. ramorum*) [23]; 3) the effector type; 4) RXLR family [23]; 5) gene environment based on intergenic distances [24]; 6) the estimated average breadth of coverage of the gene.
(XLS)

Table S16 **List of 4,934 genes of** ***P. infestans*** **and their expression profiles during infection on potato.** This list includes for each of the 4,934 coding genes: 1) annotations [23]; 2) the presence of secretion signals [23], [58]; 2) whether they belong to the 1∶1∶1 *Phytophthora* spp. core orthologs (*P. infestans: P. sojae: P. ramorum*) [23]; 3) the effector type; 4) RXLR family [23]; 5) gene environment based on intergenic distances [24]; 6) if there is gene induction in potato.
(XLS)
