# Supplementary material for: Genome Analyses of an Aggressive and Invasive Lineage of the Irish Potato Famine Pathogen
Source: PLoS Pathog. 2012 Oct 4;8(10):e1002940. doi: 10.1371/journal.ppat.1002940 (PMC3464212; doi:10.1371/journal.ppat.1002940)
Supplement: Text S1 — Supplementary Figures S1–S21. (PDF) [file ppat.1002940.s001.pdf]

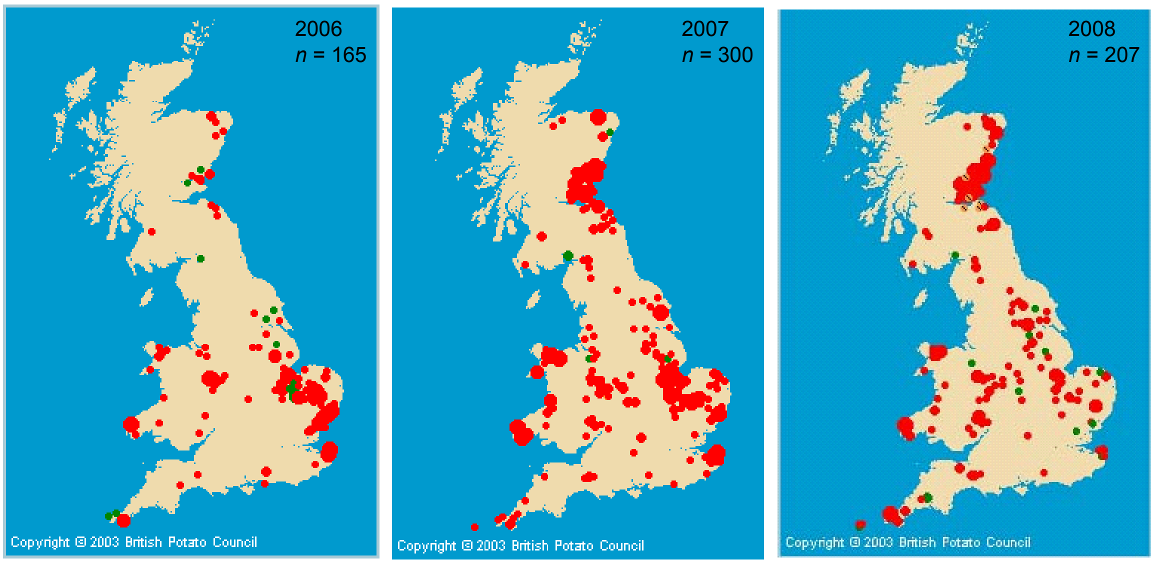

Figure S1 Location of sampled potato late blight outbreaks in Great Britain (GB) over the course of the 2006-2008 seasons as part of the Potato Council fight against blight campaign. The number of outbreaks (*n*) is shown on each map. (Figures courtesy of Potato Council).

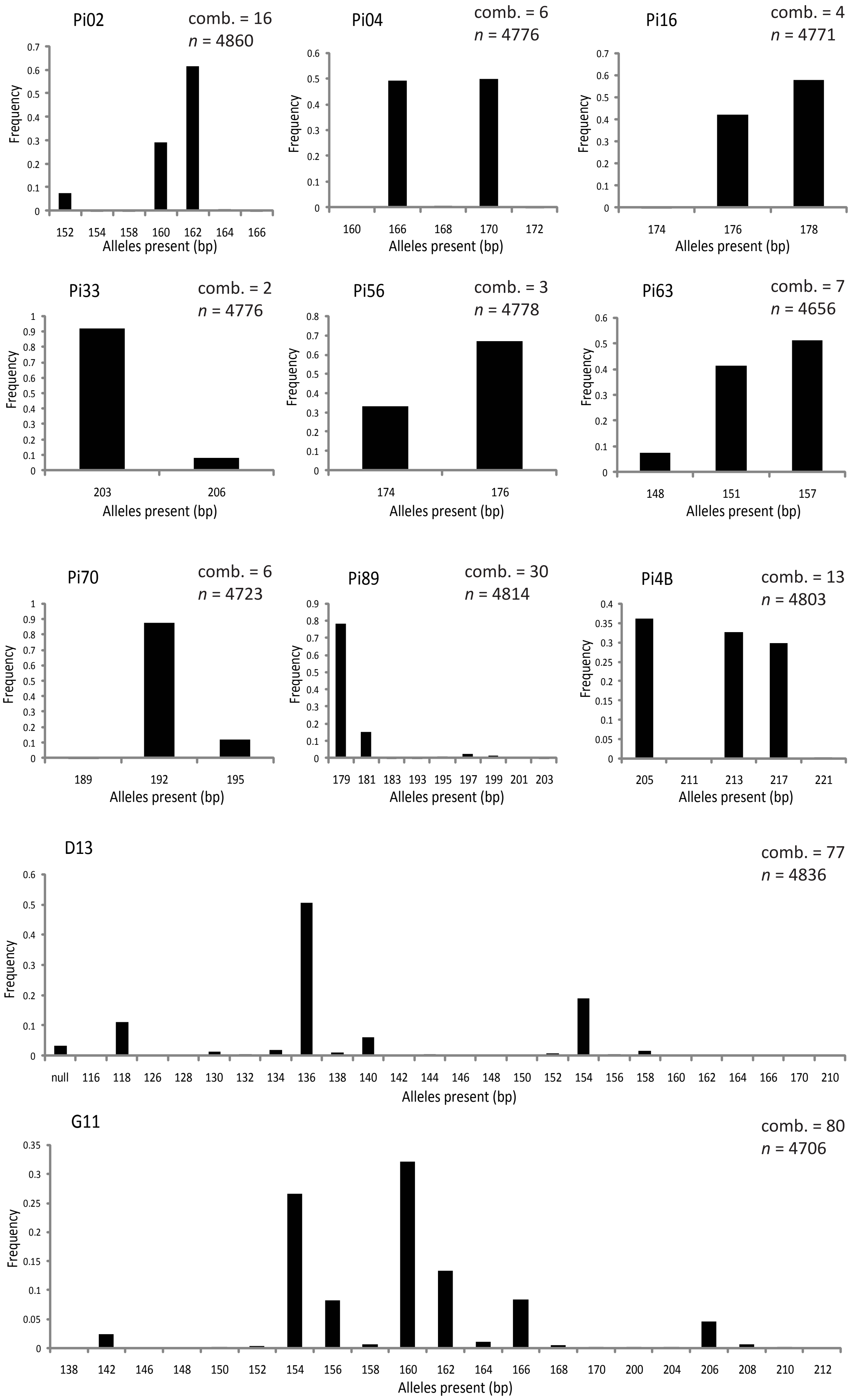

Figure S2 Allele frequency recorded at each of 11 SSR loci amongst Great Britain (GB) isolates of *P. infestans* genotyped in this study. Note that only alleles that were recorded are displayed on the x-axis but the bars of those at very low frequencies are not visible. For each locus the number of allele combinations and the number of isolates *(n)* are shown. Only at locus D13 were null alleles recorded.

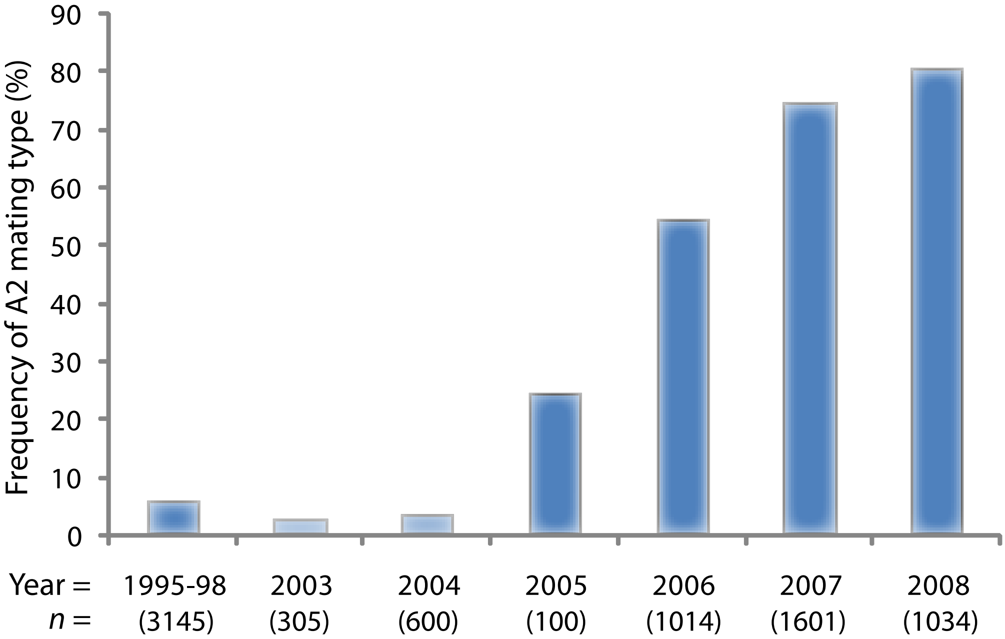

Figure S3 Change in *P. infestans* A2 mating type frequency over time. The data for 1995-98 is from previously published studies [2, 3] and all other data from the current study. Number of isolates examined *(n)* is shown beneath each bar.

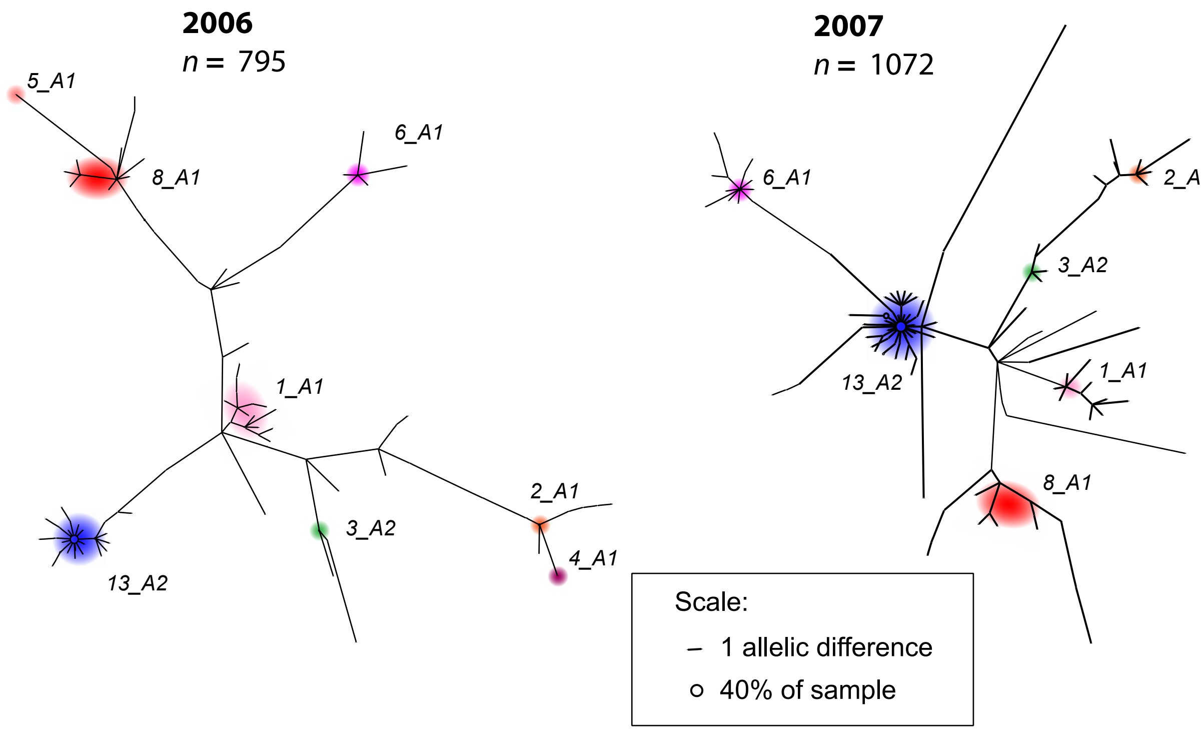

Figure S4 Minimum spanning trees showing the genetic distance among *P. infestans* multilocus genotypes (MLGs) sampled in 2006 and 2007. The size of the node is proportional to the genotype frequency in that year. Distances between nodes are proportional to genetic distance. Each tree is one of many tied trees and connections between nodes are not meant to suggest mutational relationships. The nodes of the main MLGs are coloured and named according to Figure 1 and Table S2 inText S2.

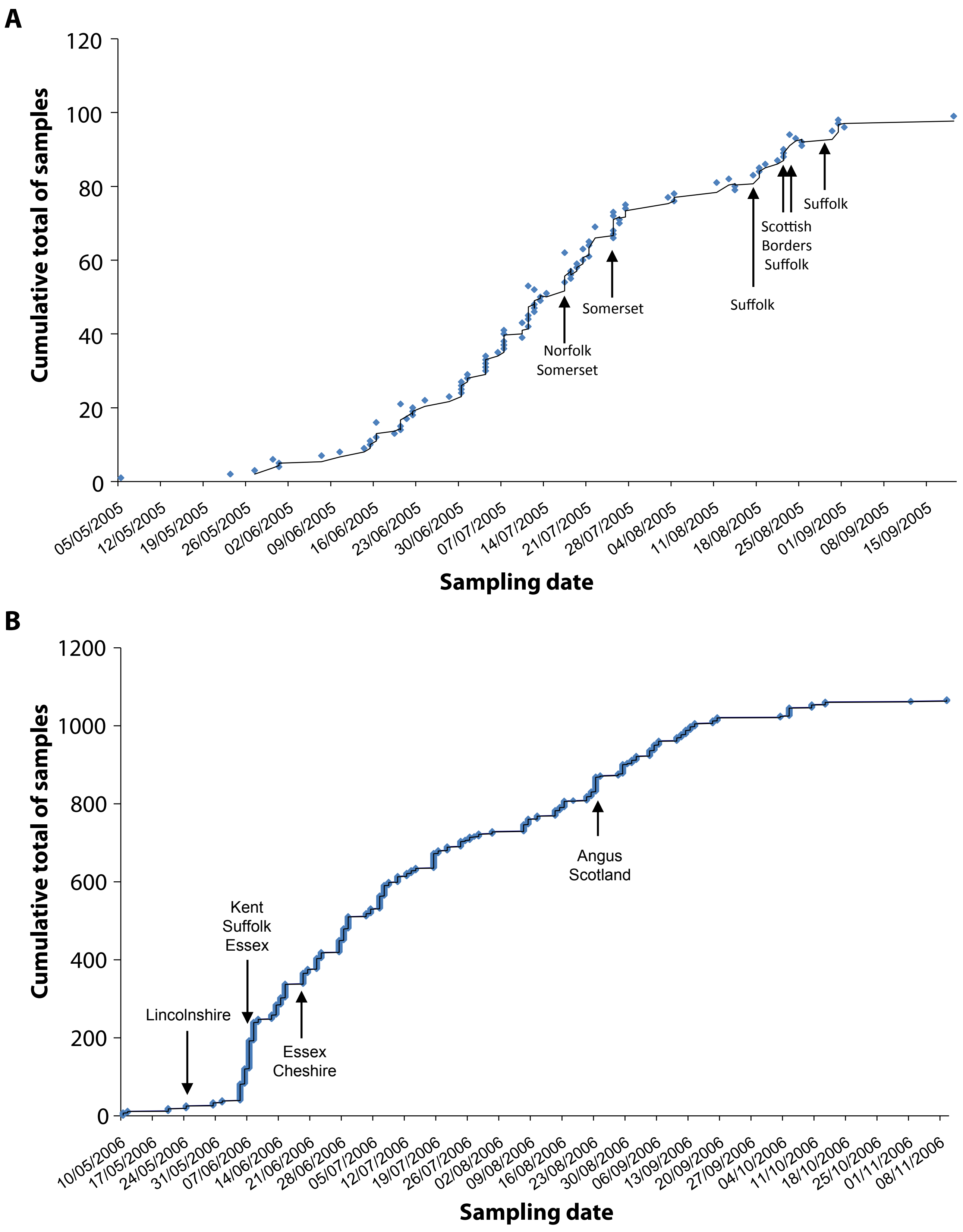

Figure S5 Dates of the first recorded MLG *13_A2* outbreaks. Cumulative total sample numbers plotted by arrival date in (A) 2005 and (B) 2006. The first recorded outbreaks in which MLG *13_A2* was found and their GB locations are marked with arrows. All the locations are counties in England except Angus and Scottish borders which are in Scotland.

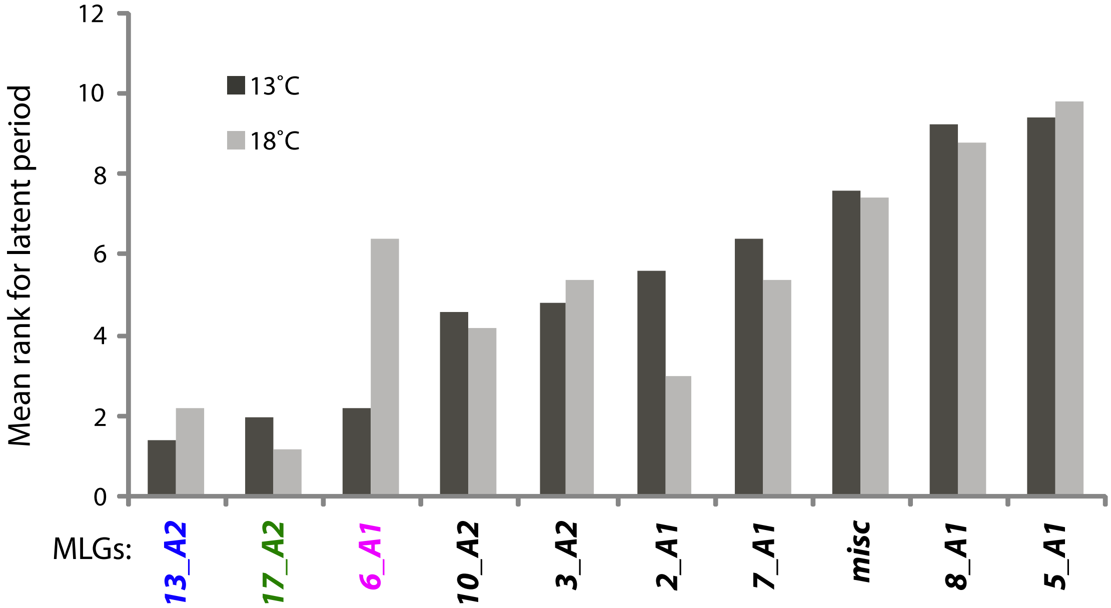

Figure S6 Mean rank of latent period scores of 10 *P. infestans* multilocus genotypes (MLGs) at 13°C and 18°C in five potato cultivars. MLG *13_A2* resulted in short latent periods at both 13°C and 18°C whereas MLG *6_A1* had amongst the shortest latent periods at 13°C but on average ranked only sixth at 18°C.

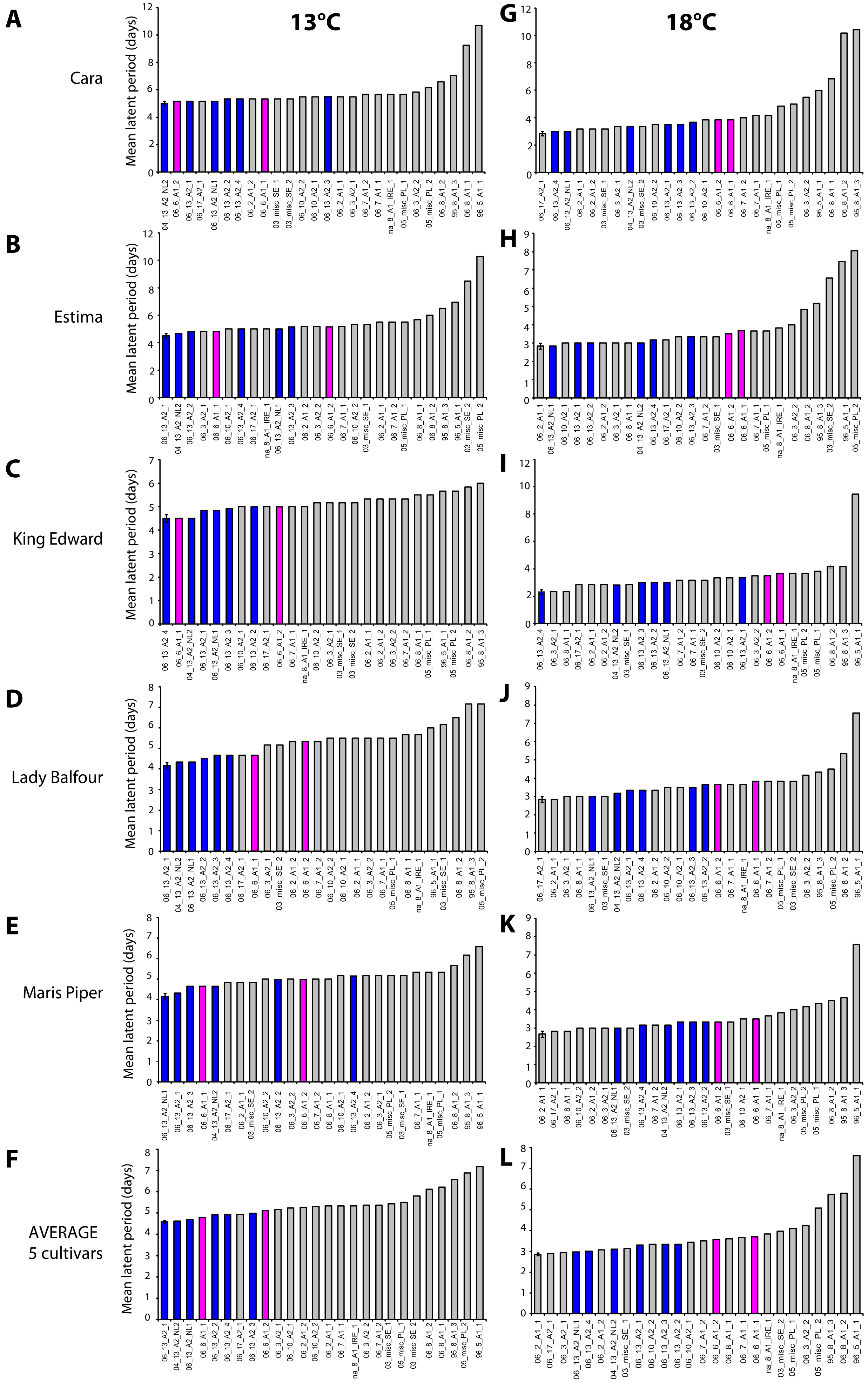

Figure S7. Mean latent period of *P. infestans* multilocus genotypes (MLGs) on leaflets of five potato cultivars at 13°C and 18°C. (A to F) leaflets incubated at 13°C or (G to L) 18°C. In each case isolates are ranked by mean latent period duration and labeled by year of isolation and MLG, with MLG *13_A2* and *6_A1* shaded in blue and pink respectively. Bars show the s.e.m = 0.3171 d.f. 613 (A to E); s.e.m = 0.3265 d.f. 540 (G to K). Average of periods on 5 cultivars shown in F and L, with bars showing the s.e.m. = 0.1412 d.f. 597 (F); s.e.m. = 0.1414 d.f. 595 (L).

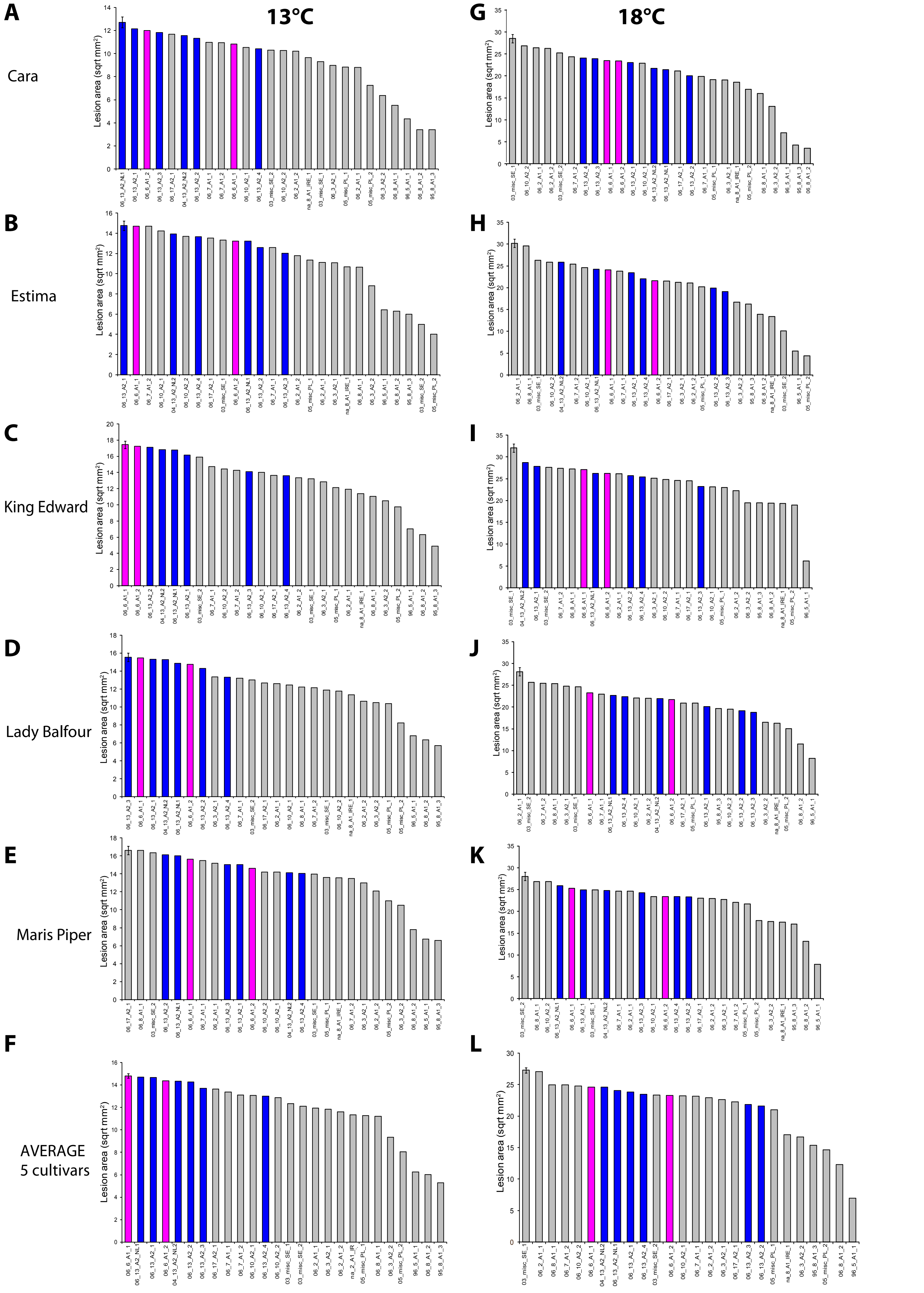

Figure S8. Mean lesion area of *P. infestans* multilocus genotypes (MLGs) on leaflets of five potato cultivars at 13°C and 18°C. (A to F) leaflets incubated at 13°C (G to L) or 18°C. In each case isolates are ranked by mean lesion size and labeled by year of isolation and MLG, with MLG *13_A2* and *6_A1* shaded in blue and pink respectively. Bars show the s.e.m = 0.4064 d.f. 624 (A to E); s.e.m = 0.0823 d.f. 619 (G to K). Average of areas on 5 cultivars shown in F and L, with bars showing the s.e.m. = 0.3171 d.f. 613 (F); s.e.m. = 1.902 d.f. 552 (L).

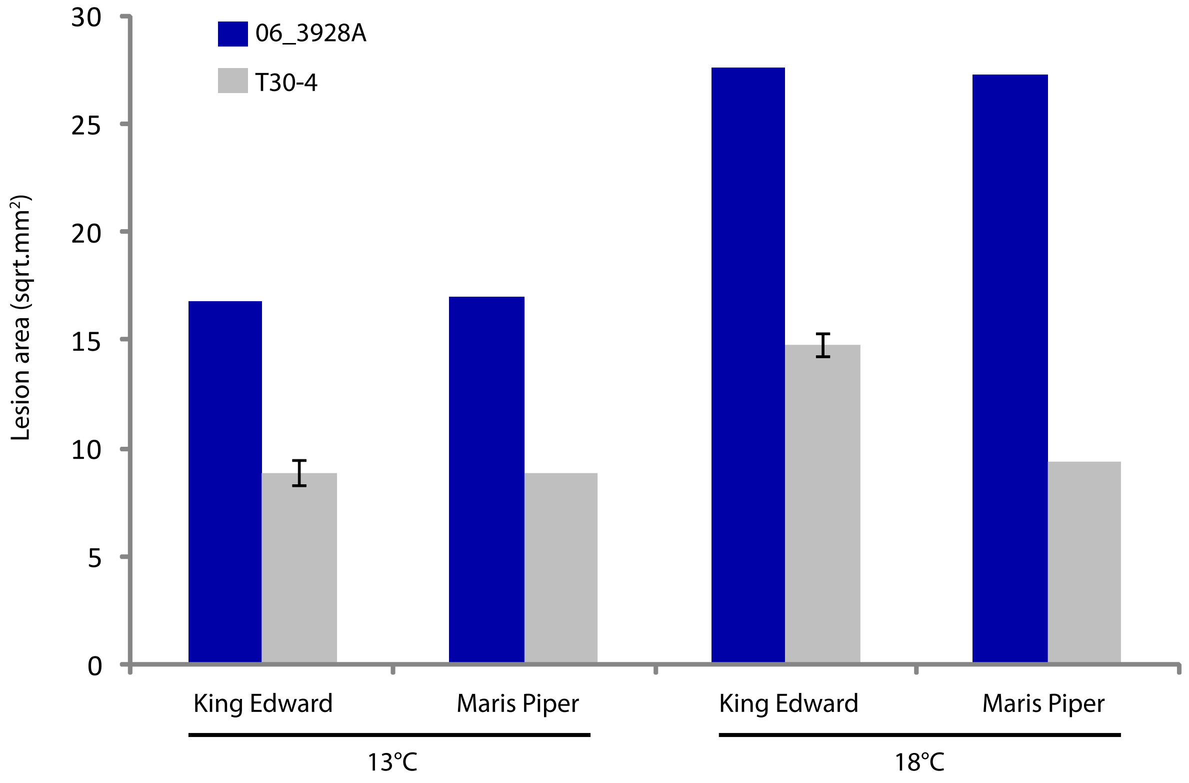

Figure S9 Mean lesion area on leaves of potato cultivars King Edward and Maris Piper caused by *P. infestans* 06_3928A and T30-4 strains incubated at 13°C and 18°C. Lesions were measured at 6 days post inoculation (dpi). Error bars show s.e.m. of 1.127 d.f. 115 at 13°C and 1.162 d.f. 119 at 18°C.

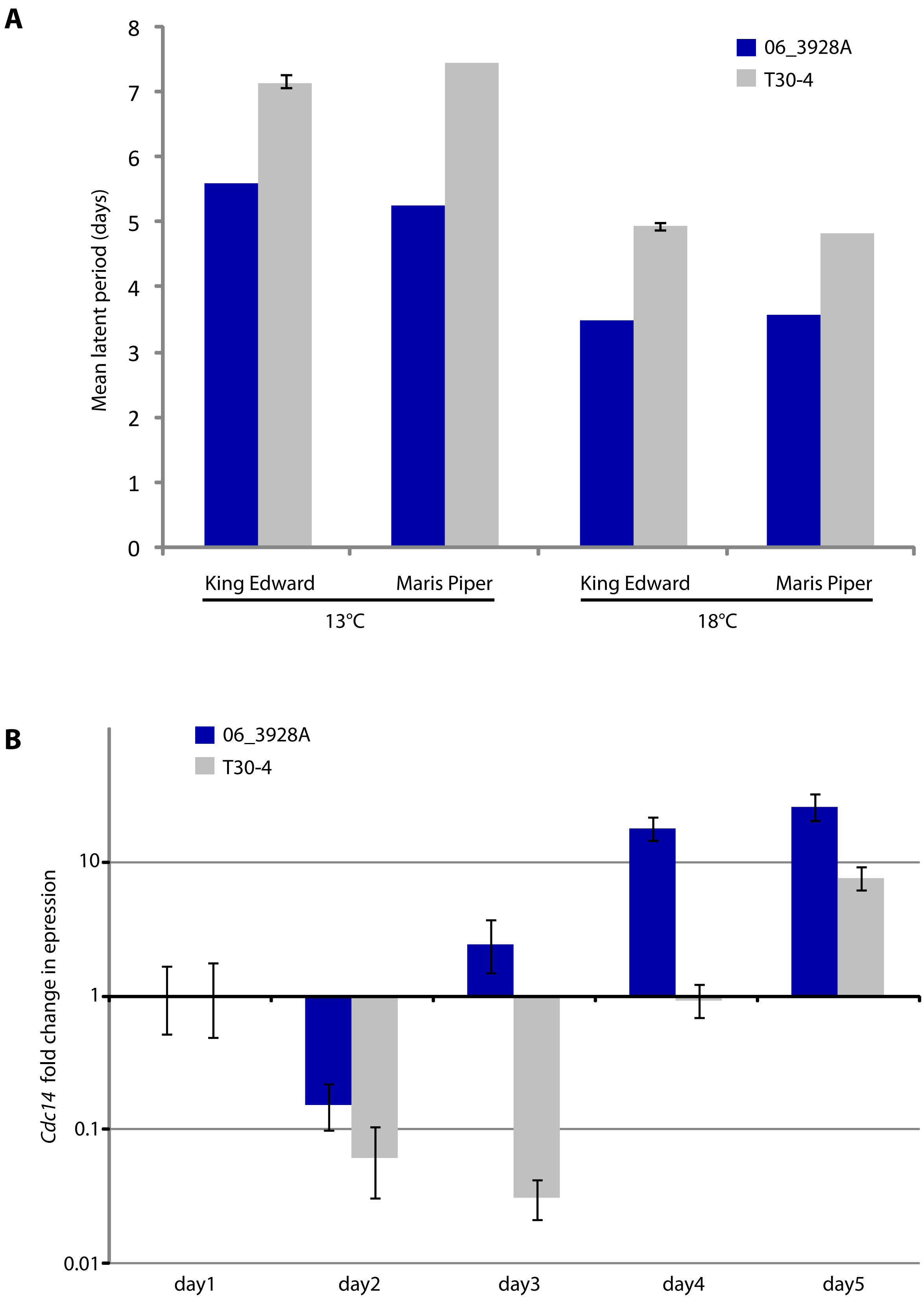

Figure S10 Differences in latent period on potato between *P. infestans* strains 06_3928A and T30-4 correlates with differences in *Cdc14* gene induction during the biotrophic phase of infection. (A) Mean latent period scored on leaves of potato cultivars King Edward and Maris Piper was 1.2 (13°C) to 2.2 (18°C) day shorter for 06_3928A than T30-4. Error bars show s.e.m. of 0.2 d.f. 87 at 13°C and 0.1 d.f. 97 at 18°C. (B) Expression of *P. infestans Cdc14* gene measured by QRT-PCR in a time course infection on susceptible potato *cv* Desiree incubated at 18°C. The graph shows that *Cdc14* was induced during the biotrophic phase of infection in 06-3928A (blue) but not in T30-4 (grey). Data presented on the Y-axis correspond to the fold change relative to normalized expression at day 1 after inoculation (given a value of 1) for each individual *P. infestans* strain calculated by the ΔΔCt method with error bars representing +/- s.e.

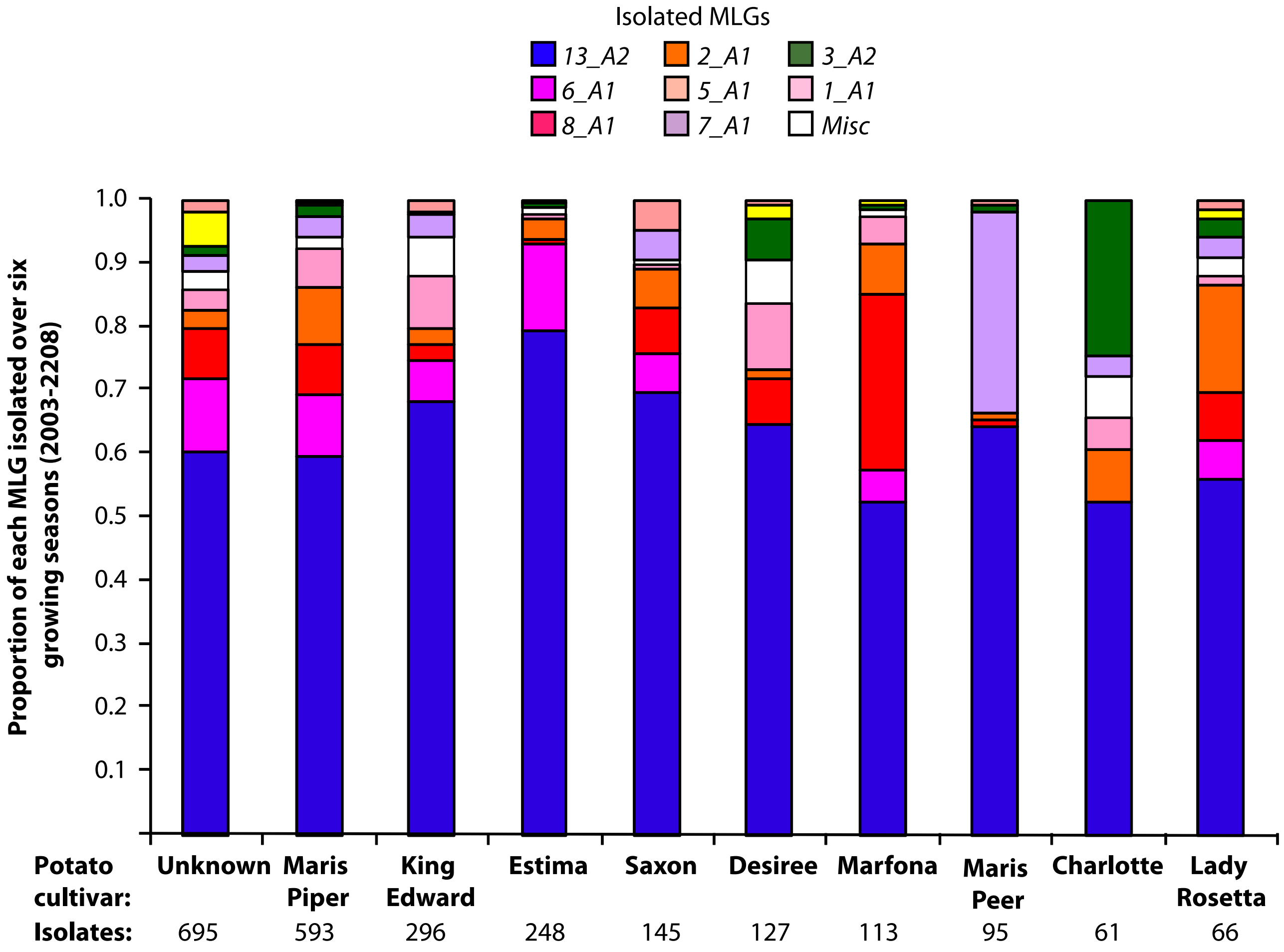

Figure S11 The proportion of each *P. infestans* multilocus genotypes (MLGs) isolated from the ten most sampled potato cultivars over six growing seasons from 2003-2008. The numbers within each column refer to the MLG names and those beneath each column to the number of isolates collected on that cultivar.

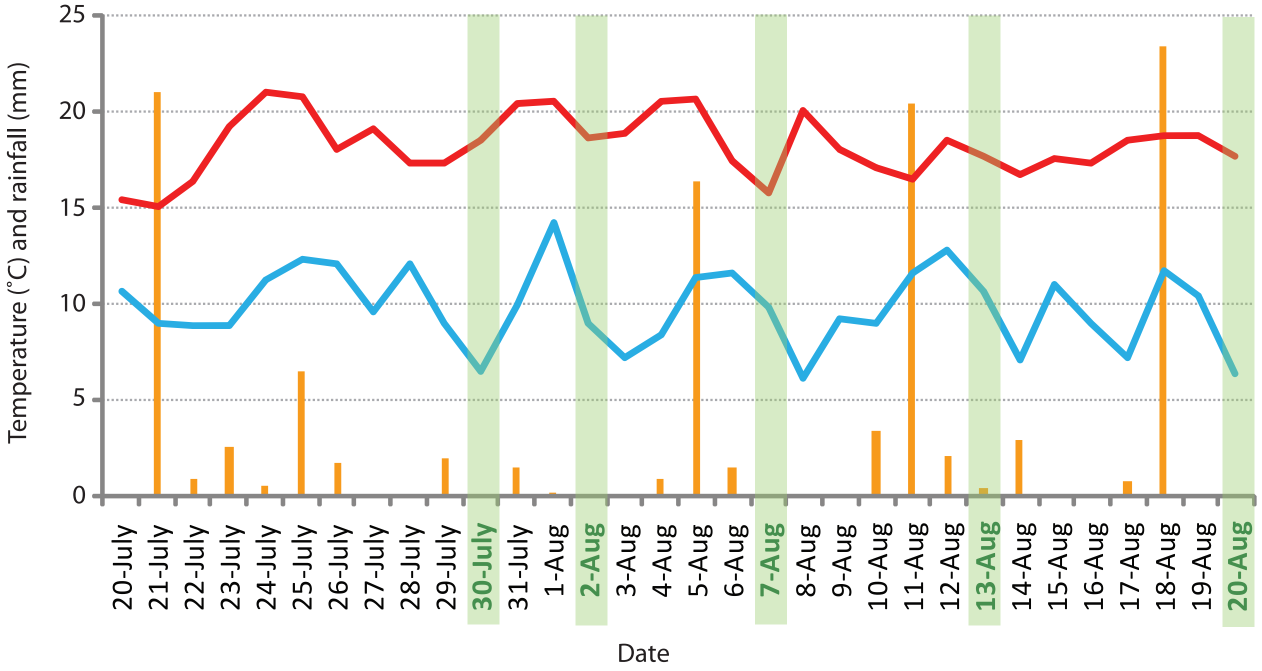

Figure S12 Daily minimum (blue line) and maximum (red line) temperatures (°C) and rainfall (orange bars - mm rain per day) during the mark-and-recapture aggressiveness trial. Sampling dates are highlighted and shown in bold green text.

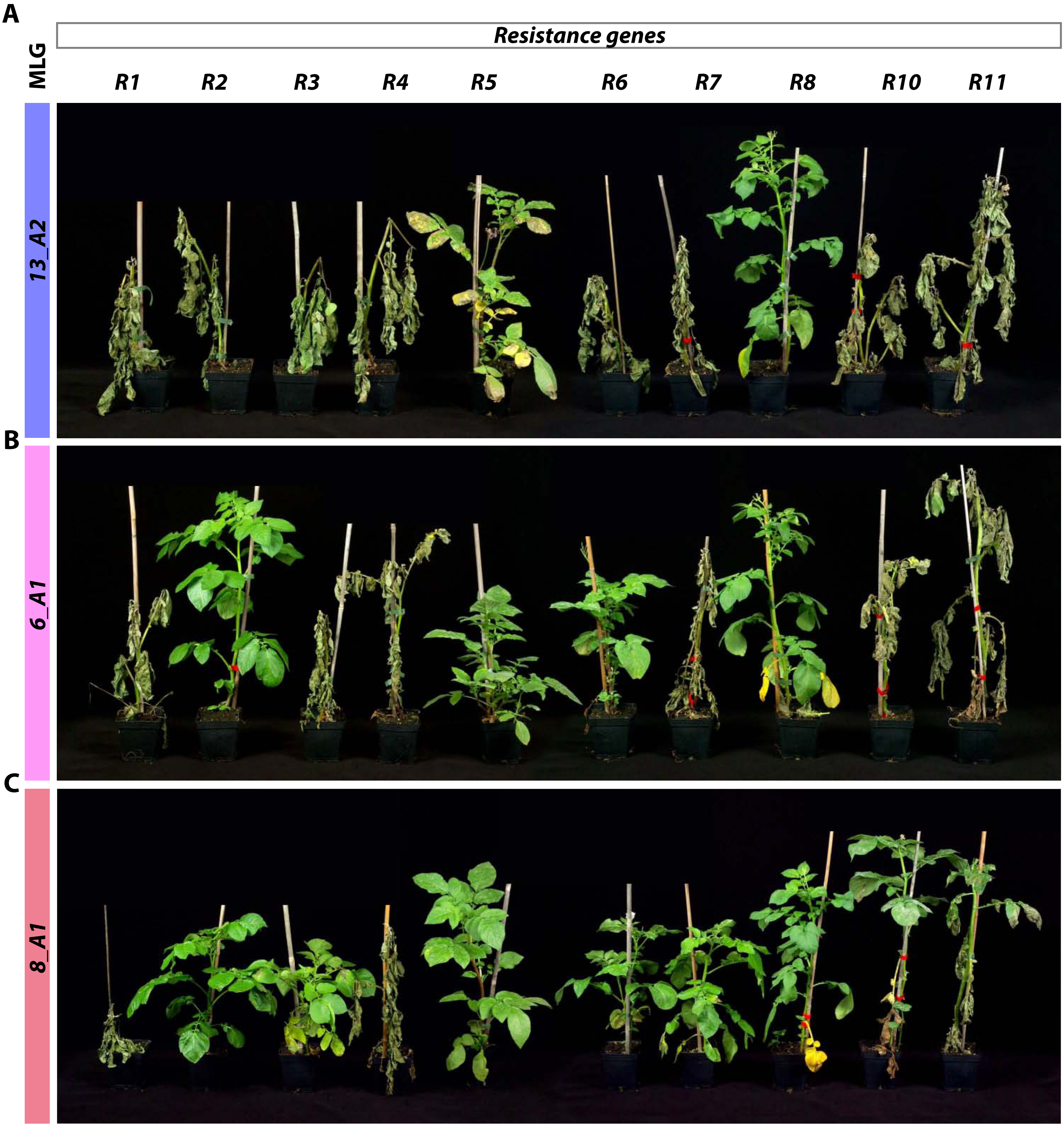

Figure S13 Virulence profiles of three *P. infestans* multilocus genotypes (MLGs): *13_A2, 6_A1* and *8_A1*. Plants of Black's differential *R* gene series (excluding *R9*) at 7 days post inoculation with (A) isolate 06_3928A (MLG *13_A2*, virulence 1, 2, 3, 4, 5, 6, 7, 10, 11), (B) isolate 06_4100A (MLG *6_A1*, virulence 1, 3, 4, 7, 10, 11), (C) isolate 06_4256B (MLG *8_A1*, virulence 1, 4, 7, 11). Another eight isolates of MLG *13_A2* had an identical virulence profile to that shown. Plants of the *R9* differential were not available in this test but were fully resistant in all subsequent tests of these and other isolates of the same MLG (data not shown).

A

Min Depth	% Cons.	False Positives (%)	True Positives (%)
3	80	0.01309	89.40497
4	80	0.01306	89.08481
5	80	0.01301	88.71160
6	80	0.01297	88.35726
7	80	0.01295	88.03096
8	80	0.01293	87.71908
9	80	0.01291	87.39771
10	80	0.01290	87.04936
12	80	0.01285	86.41784
14	80	0.01279	85.79365
16	80	0.01275	85.24296
18	80	0.01265	84.62325
20	80	0.01261	84.03207
22	80	0.01256	83.46555
24	80	0.01252	82.93276
26	80	0.01248	82.36322
28	80	0.01242	81.77808
30	80	0.01237	81.25368
3	90	0.01255	87.76172
4	90	0.01251	87.44224
5	90	0.01249	87.14974
6	90	0.01247	86.86975
7	90	0.01245	86.62821
8	90	0.01242	86.38521
9	90	0.01241	86.14026
10	90	0.01240	85.82179
12	90	0.01236	85.25586
14	90	0.01231	84.68314
16	90	0.01229	84.18760
18	90	0.01221	83.63598
20	90	0.01217	83.09963
22	90	0.01214	82.56850
24	90	0.01211	82.05986
26	90	0.01207	81.51310
28	90	0.01202	80.96014
30	90	0.01197	80.46071
3	95	0.01192	82.66312
4	95	0.01189	82.34517
5	95	0.01186	82.05394
6	95	0.01184	81.77511
7	95	0.01183	81.53478
8	95	0.01180	81.29285
9	95	0.01179	81.04901
10	95	0.01178	80.79072
12	95	0.01174	80.34777
14	95	0.01170	79.91448
16	95	0.01169	79.56508
18	95	0.01162	79.15060
20	95	0.01159	78.73451
22	95	0.01156	78.26415
24	95	0.01153	77.80932
26	95	0.01149	77.31357
28	95	0.01144	76.80926
30	95	0.01140	76.36571

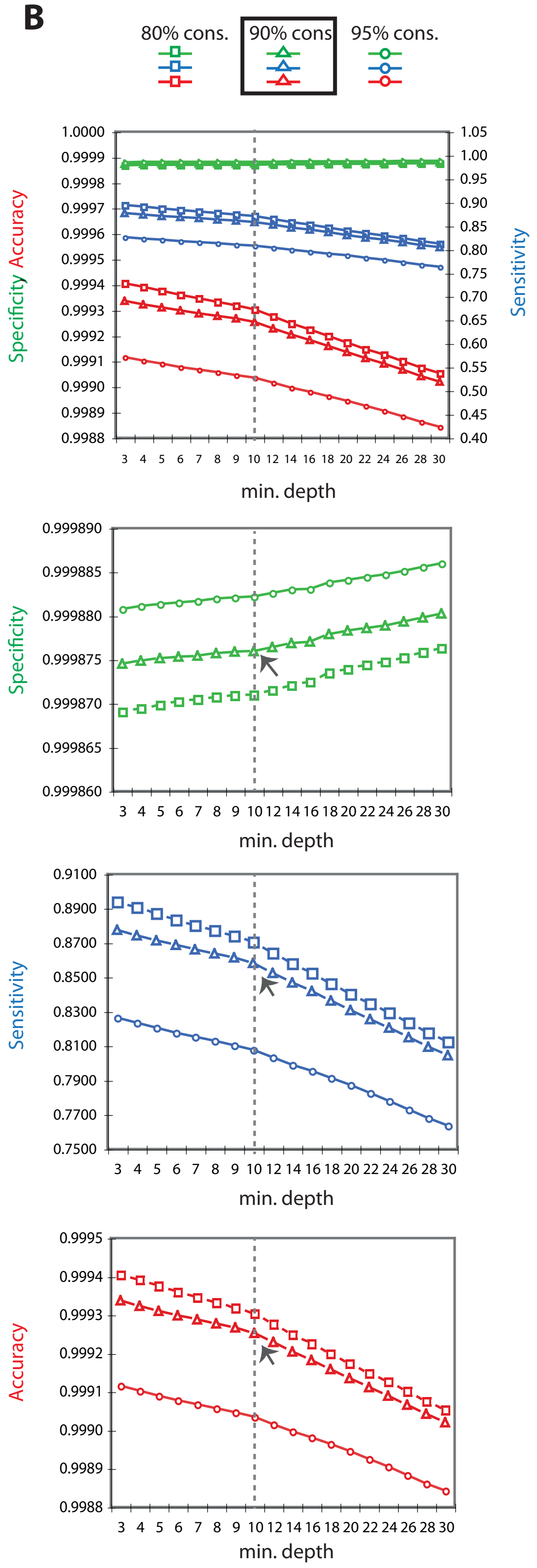

Figure S14 False Discovery Rate (FDR) analysis in *P. infestans 13_A2* isolate 06_3928A. (A) Summary of false and true positive rates obtained with 54 parameter sets in the sequenced genome of *P. infestans 13_A2* isolate 06_3928A. Percentage of consensus bases chosen is presented with the black square box. Arrow point the minimum read depth chosen. (B) Accuracy, Specificity and Sensitivity of FDR test obtained with 54 parameter sets tested in 58x coverage genome. Dotted line highlights parameter set chosen for subsequent analyses. Arrow in (A) indicates the point that defines the minimum read depth that allows detecting SNPs with 99.9% of accuracy and 85.8% of sensitivity.

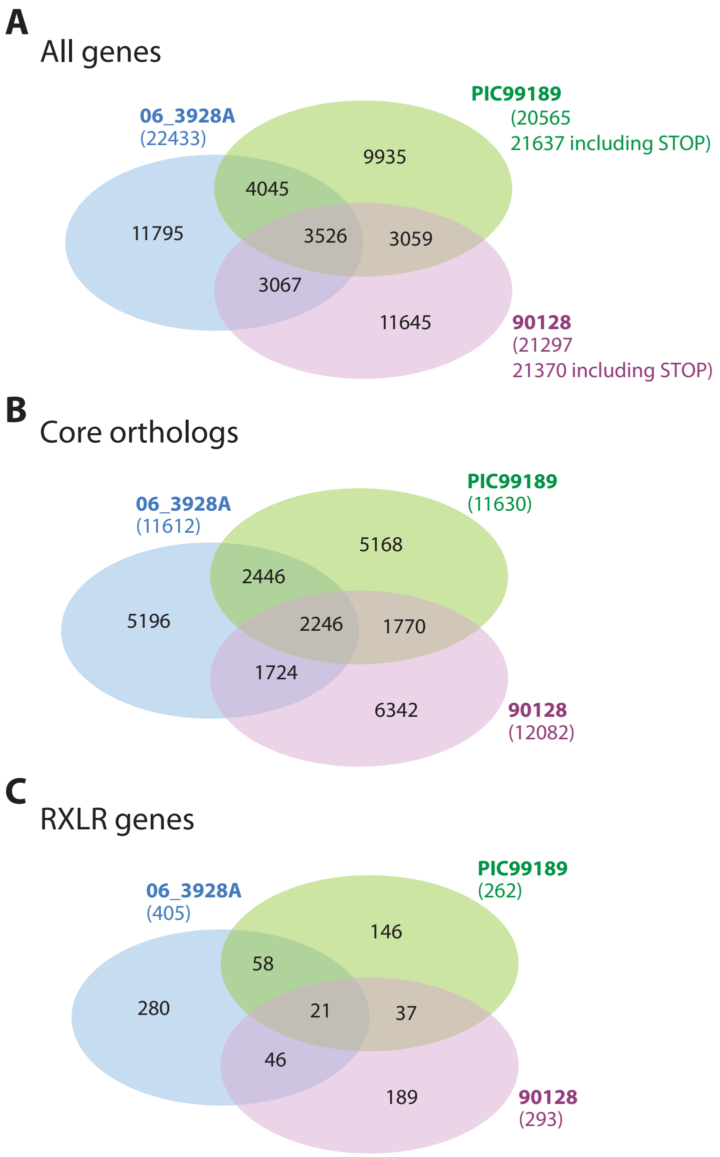

Figure S15 Venn diagrams with number of SNPs in coding genes in three sequenced *P. infestans* isolates. SNPs in the *P. infestans 13_A2* isolate 06_3928A were called in positions with 90% of consensus bases and a minimum read depth of 10. SNPs in the *P. infestans* PIC99189 and 90128 isolates were called as reported [23]. SNPs causing loss of stop codons were excluded for each category. Total numbers of SNPs in each isolate are indicate in parenthesis. (A) Total number of SNPs in all coding genes. (B) Total number of SNPs in core orthologs genes. (C) Total number of SNPs in secreted RXLR effector genes.

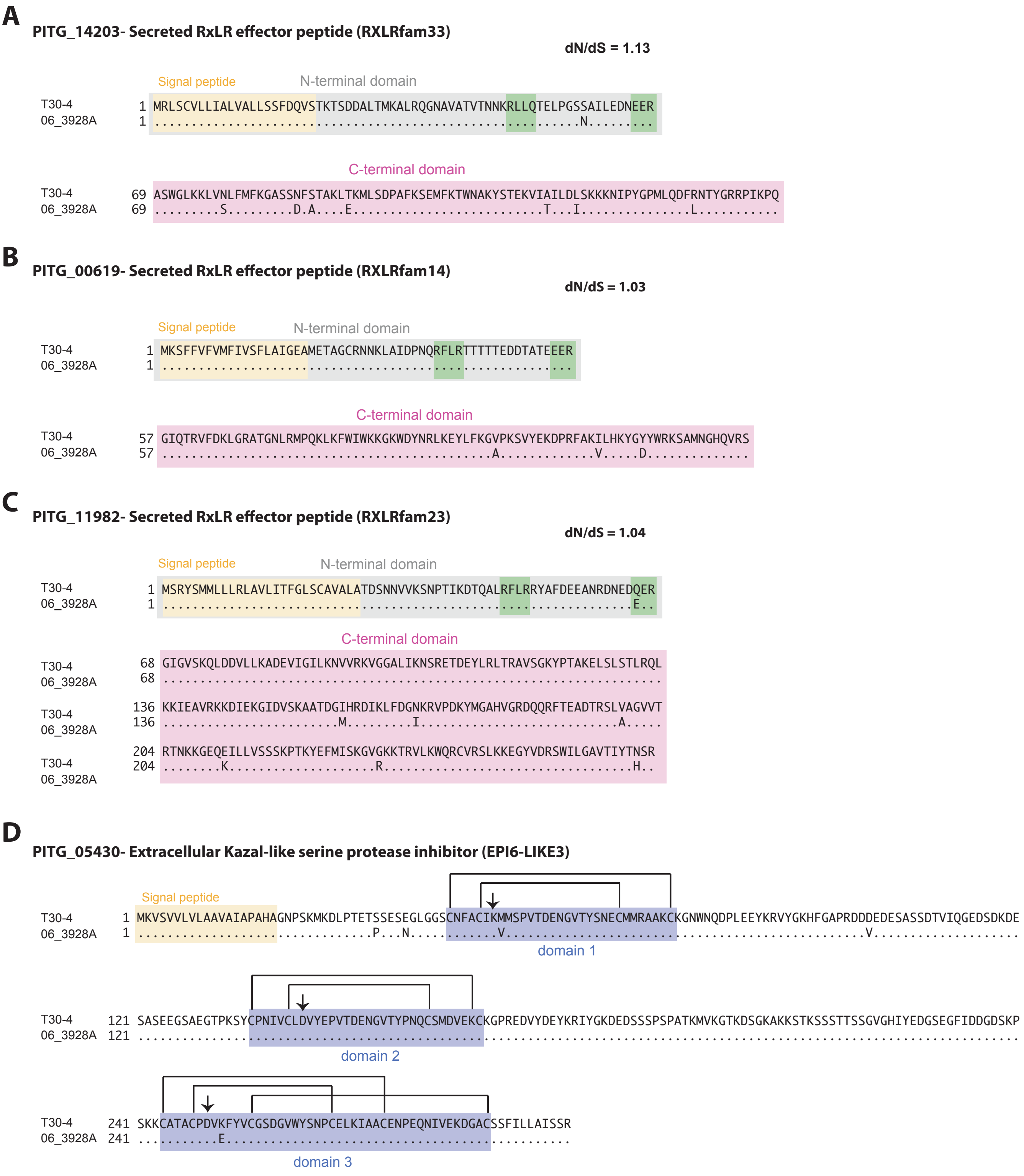

Figure S16 Examples of effectors showing dN/dS ratios>1 in the sequenced *P. infestans 13_A2* isolate 06_3928A compared to T30-4. Alignment of protein sequences from effectors of *P. infestans* T30-4 (top) and 06_3928A (bottom) where differences in amino acids between both sequences are indicated. (A) PITG_14203 secreted RXLR effector with dN/dS ratio of 1.13. (B) PITG_00619 secreted RXLR effector with dN/dS ratio of 1.03. (C) PITG_11952 secreted RXLR effector with dN/dS ratio of 1.04. (D) PITG_05430 extracellular Kazal-like serine protease inhibitor effector with dN/dS ratio of 1.20. N-terminal domains are shown in grey, signal peptide sequences are shown in yellow, RXLR-EER motifs are shown in green, C-terminal effector domains are shown in pink, Kazal-like domains are shown in blue and P1 amino acid residues within the Kazal-like domains are indicated with an arrow.

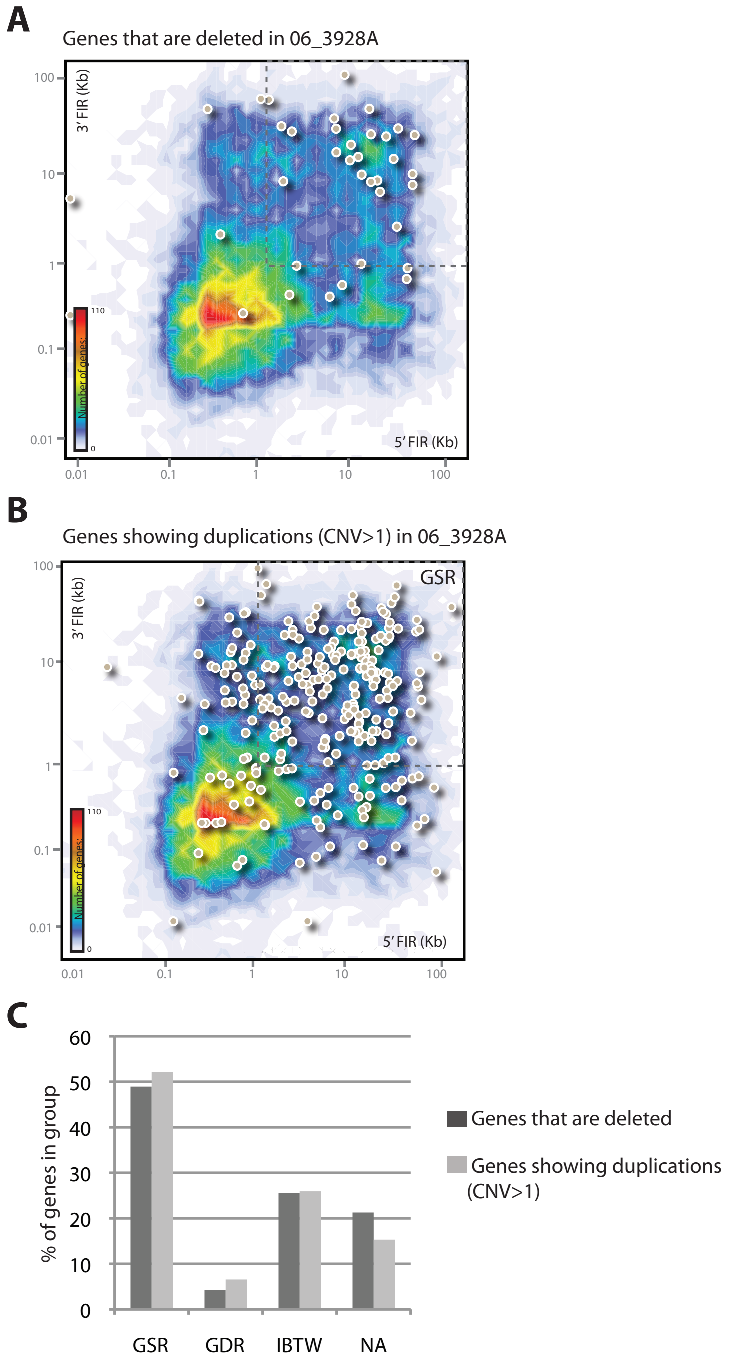

Figure S17 P. infestans T30-4 homologs of genes showing copy number variations (deletions and duplications) in 06_3928A isolate are frequently located in gene sparse regions (GSRs). (A) Gene density plot showing the location of T30-4 homologs of genes missing in 06_3928A based on their intergenic distances in the P. infestans reference genome strain T30-4 [33]. (B) Gene density plot showing the location of T30-4 homologs of genes with duplications (CNV>1) in 06_3928A based on their intergenic distances in the P. infestans reference genome strain T30-4. (C) Distribution of T30-4 homologs of 06_3928A genes in genome regions of P. infestans reference genome strain T30-4. Y-axis indicates percentages of genes in the genome region category. X-axis indicates genome region category classified according to the flanking intergenic distance (FIR) length (Kb) at the 5' and 3' ends [23]. A gene located in the gene sparse region (GSR) shows 5' and 3'FIR>1.5 Kb. A gene located in the gene dense region (GDR) shows 5' and 3'FIR<1.5 Kb. A gene located in the in-between region (IBTW) shows 5' FIR>1.5 but 3'FIR<1.5 Kb or vice versa. A gene where the region could not be calculated is named as not applicable (NA).

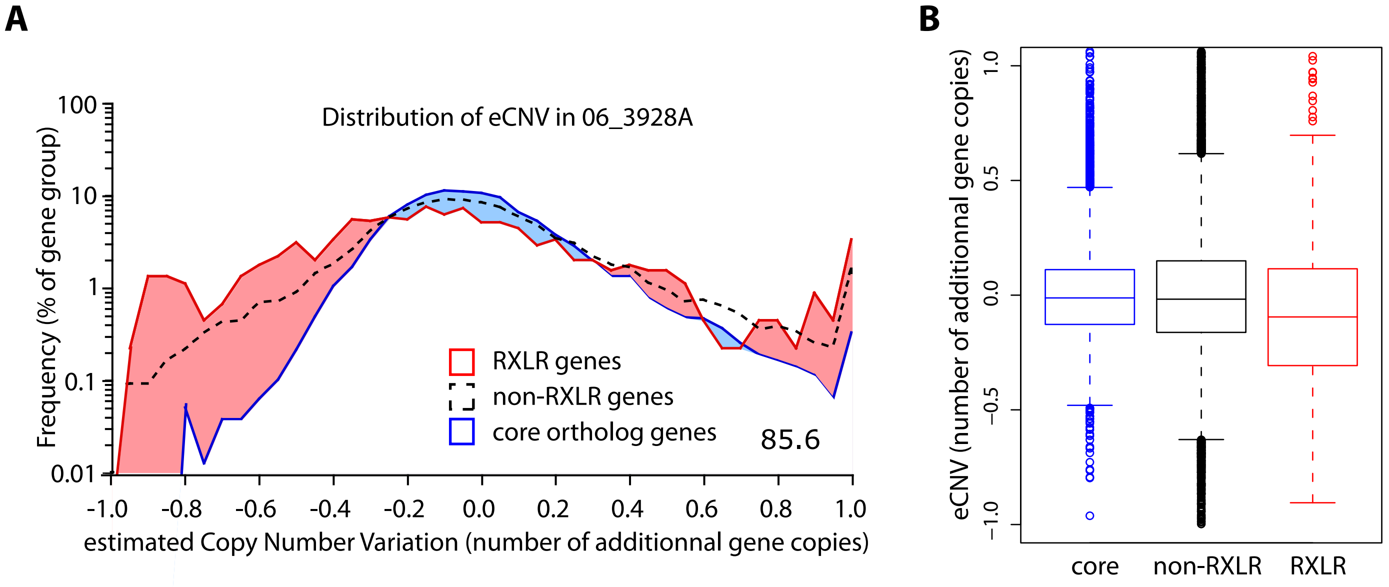

Figure S18 Distribution of polymorphism in genes of *P. infestans 13_A2* isolate 06_3928A. Y-axis indicates of estimated copy number variation (eCNV) in (A) given for RXLR genes, other (non-RXLR) genes and core ortholog genes [15]. Y-axis shows the estimated number of additional gene copies (eCNV) in (B) given for RXLR genes, other (non-RXLR) genes and core ortholog genes. Box and whisker plots in (B) show median, first and third quartile, and first values beyond 1.5 times the interquatile range.

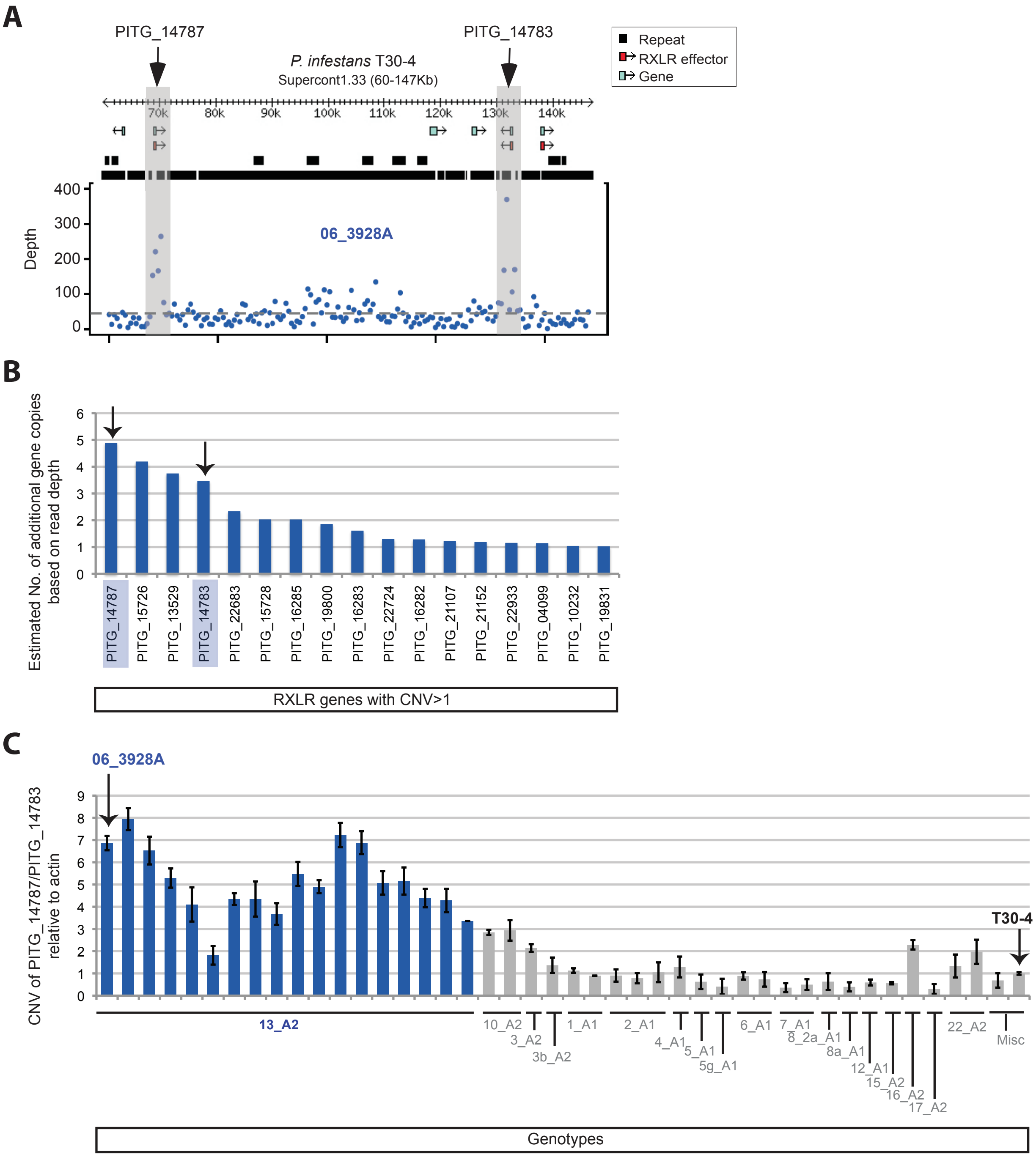

Figure S19 Prediction and validation of duplication events found in two RXLR genes of the sequenced P. infestans 13_A2 isolate 06_3928A. (A) Depth of coverage plot showing duplication events in PITG_14787 and PITG_14783 RXLR genes in 06_3928A isolate. 70 Kb genomic region from supercont1.33 containing both RXLR genes was scanned with a window size of 500 bp. Region where sequence reads from 06_3928A aligned to PITG_14787 or PITG_14783 genes are highlighted within grey vertical bars. Dashed grey lines indicate the genome average depth of coverage. (B) Histogram showing the top 17 RXLR genes from 06_3928A isolate having at least one additional gene copy compared to T30-4. Black arrows point to two RXLR genes with CNV>1: PITG_14787 and PITG_14783 also highlighted in blue. (C) Histogram showing PCR validation of CNV events in PITG_14787 and PITG_14783 RXLR genes. Black arrows point the sequenced isolate 06_3928A and the reference genome strain T30-4.

A

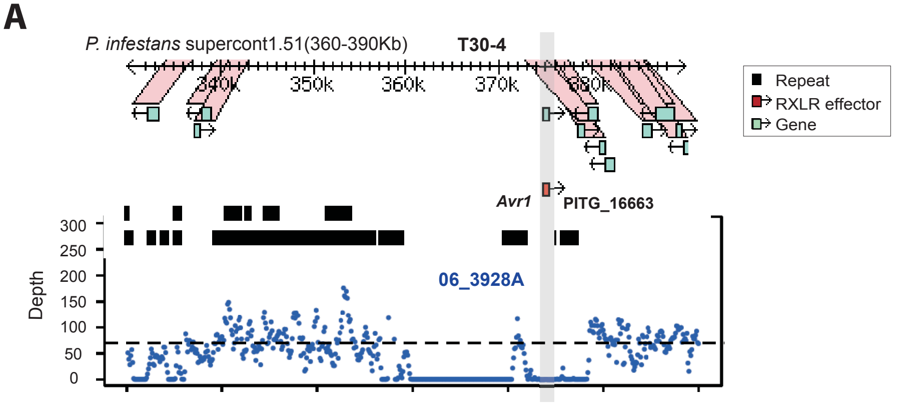

B

P. infestans strain	MLG	PCR product with *Avr1* primers
→ **T30-4 SW**	**Misc**	**+**
→ **2006_3928A**	**13_A2**	**-**
2006_3964A	13_A2	-
2006_4132B	13_A2	-
2006_3884B	13_A2	-

Figure S20 Prediction and validation of *Avr1* deletion in the sequenced *P. infestans 13_A2* isolate 06_3928A. (A) Plot of sequencing depth of coverage of Illumina reads from 06_3928A aligned to the region of supercontig1.51 from T30-4 strain containing the avirulence effector *Avr1* (PITG_16663) (in red) scanned with a window size of 200 bp. Region where sequence reads from 06_3928A aligned to *Avr1* gene is highlighted within grey vertical bars. Dashed grey lines indicate the genome average depth of coverage. Note the ~18 Kb sub-region (from 361 to 379 Kb) that shows reduced coverage in reads from isolate 06_3928A indicating high sequence divergence in this isolate. (B) Table showing the results of the PCR validation of *Avr1* deletion in various *P. infestans* strains. Black arrows point to the sequenced 06_3928A and the reference genome strain T30-4.

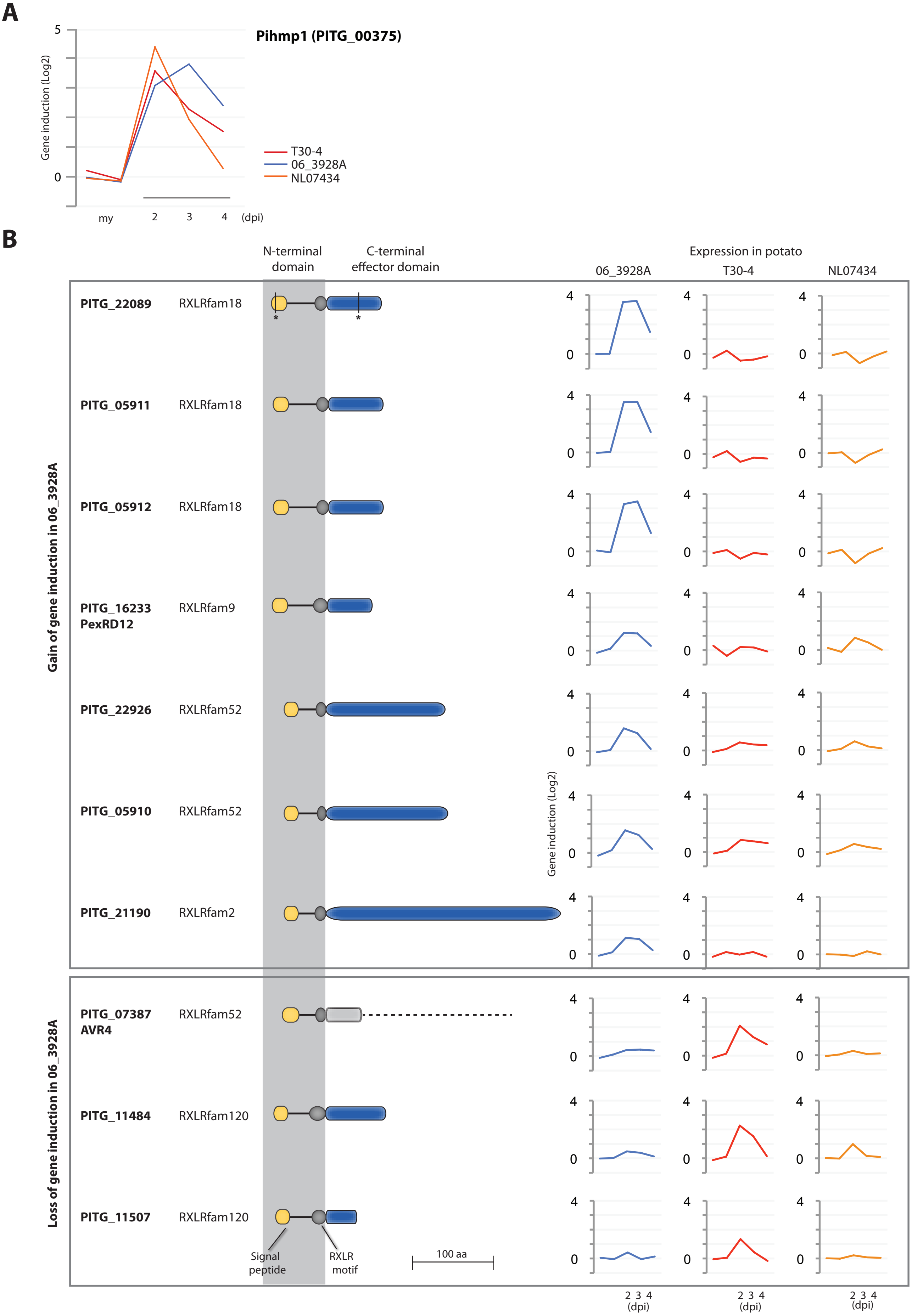

Figure S21 Expression polymorphisms in *P. infestans 13_A2* isolate 06_3928A. (A) Expression pattern of the haustorial-specific gene *Pihmp1* [32] during potato infection in the strains analyzed in this work. Gene induction (log2) was calculated for each time point of the infected material from 2 to 4 days post inoculation (dpi) using mycelia as baseline (see Text S3). The gene induction pattern follow those reported for genes that are commonly induced *in planta* in the three strains (Figure 7B) supporting the extension of the biotrophic phase during 06_3928A infection. (B) RXLR effectors showing gene expression polymorphisms in the sequenced isolate 06_3928A. *P. infestans* RXLR effectors are modular proteins with a N-terminal (signal peptide) domain, RXLR motif, and C-terminal effector domain. A vertical bar placed in line with an asterisk show a polymorphic amino acid in protein in 06_3928A relative to T30-4. The C-terminus effector domain coloured in light grey is indicative of a truncated *Avr4* in 06_3928A. Expression in potato panel illustrates a time course expression patterns of the genes during infection of potato (2-4 dpi), and includes the expression for *P. infestans* 06_3928A, NL07434 and the reference strain T30-4 with the y-axis showing gene induction.
