# Supplementary material for: Genome Analyses of an Aggressive and Invasive Lineage of the Irish Potato Famine Pathogen
Source: PLoS Pathog. 2012 Oct 4;8(10):e1002940. doi: 10.1371/journal.ppat.1002940 (PMC3464212; doi:10.1371/journal.ppat.1002940)
Supplement: Text S2 — Supplementary Tables S1–S8 and S14–S15. (DOC) [file ppat.1002940.s002.doc]

**Table S1** **Summary data of the number of outbreaks sampled and the frequency of the A1 and A2 mating types of *P. infestans* sampled across Great Britain (GB) over time.** The data for 1995-98 is from previously published studies all other data from the current study.

| **Year** | **1995-98** | **2003** | **2004** | **2005** | **2006** | **2007** | **2008** | **Total** |
| --- | --- | --- | --- | --- | --- | --- | --- | --- |
| % A2 isolates | 5.3 | 2.3 | 3.0 | 24.0 | 54.2 | 74.3 | 80.3 | 243 |
| No. of A2 isolates | 168 | 7 | 18 | 24 | 550 | 1190 | 830 | 2787 |
| No. of A1 isolates | 2977 | 298 | 582 | 76 | 464 | 411 | 204 | 5012 |
| Total isolate number | 3145 | 305 | 600 | 100 | 1014 | 1601 | 1034 | 7799 |
| Number of outbreaks sampled | 436 | 130 | 206 | 120 | 165 | 300 | 207 | 1564 |

**Table S2 Fingerprints of isolates according to RFLP with RG57 probe or SSRs.** (A) The RG57 fingerprint mtDNA haplotype and metalaxyl resistance of MLG *13_A2* isolates collected in Great Britain (GB) in 2005. (B) The combination of SSR alleles at 11 SSR loci used to define each multilocus genotype (MLG) in this study. Variation within some clonal MLGs was apparent and indicated with a sub-MLG numbering system. The seven most prevalent variants of MLG *13_A2* are shown. Such intra-MLG variation is apparent in Figure 1b. Cross-referencing to MLG codes in previous studies is indicated .

(A)

| **Isolate number** | **County** | **mtDNA** | **RG57 fingerprint** | **Metalaxyl resistance** |
| --- | --- | --- | --- | --- |
| 05_14500 | Norfolk | Ia | 1,2,8,10,13,14,17,19,20,21,22,24,25 | Resistant |
| 05_16012 | Scottish Borders | Ia | 1,2,8,10,13,14,17,19,20,21,22,24,25 | Resistant |
| 05_14473 | Somerset | Ia | 1,2,8,10,13,14,17,19,20,21,22,24,25 | Resistant |
| 05_15094 | Somerset | Ia | 1,2,8,10,13,14,17,19,20,21,22,24,25 | Resistant |
| 05_15715 | Suffolk | Ia | 1,2,8,10,13,14,17,19,20,21,22,24,25 | Resistant |
| 05_15985 | Suffolk | Ia | 1,2,8,10,13,14,17,19,20,21,22,24,25 | Resistant |
| 05_16201 | Suffolk | Ia | 1,2,8,10,13,14,17,19,20,21,22,24,25 | Resistant |


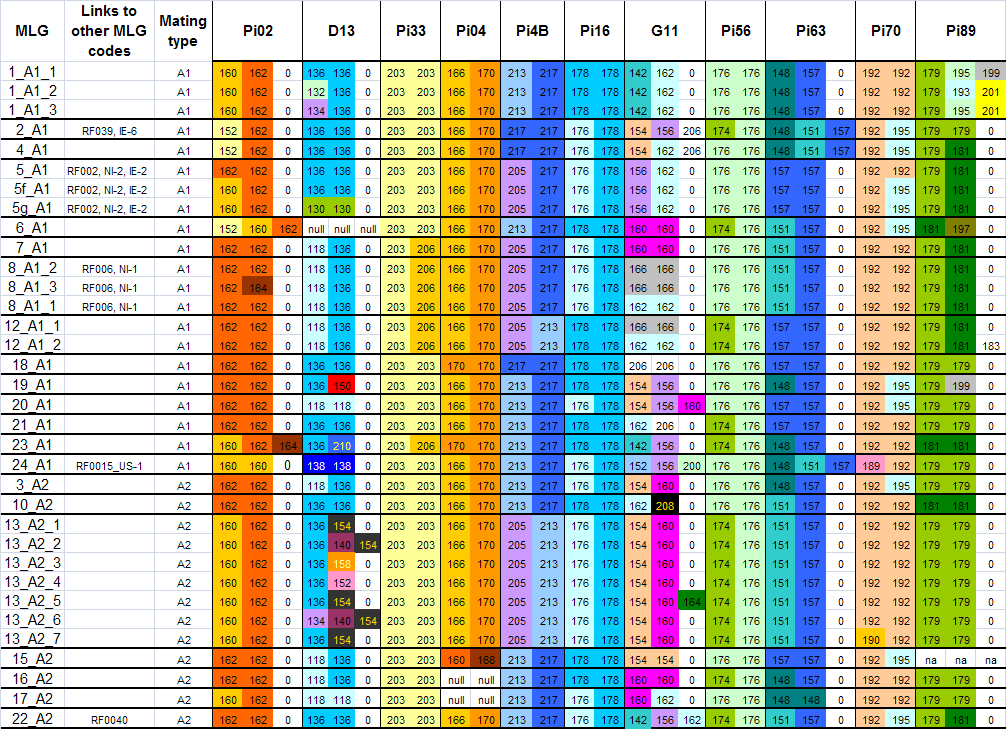
(B)

**Table S3 Number of *P. infestans* isolates of each multilocus genotype (MLG) determined in each year of this study.**

| **MLG** | **1982** | **1995** | **1996** | **1997/8** | **2003** | **2004** | **2005** | **2006** | **2007** | **2008** | **Totals** |
| --- | --- | --- | --- | --- | --- | --- | --- | --- | --- | --- | --- |
| *21_A1* | 0 | 0 | 0 | 15 | 0 | 3 | 0 | 0 | 0 | 0 | 18 |
| *4_A1* | 5 | 4 | 0 | 18 | 2 | 10 | 0 | 2 | 3 | 5 | 49 |
| *12_A1* | 0 | 0 | 0 | 0 | 3 | 6 | 5 | 6 | 11 | 1 | 32 |
| *20_A1* | 0 | 0 | 0 | 0 | 0 | 0 | 0 | 6 | 0 | 0 | 6 |
| *19_A1* | 0 | 0 | 0 | 0 | 0 | 0 | 0 | 6 | 0 | 0 | 6 |
| *5_A1* | 0 | 30 | 47 | 25 | 33 | 93 | 4 | 8 | 8 | 7 | 255 |
| *18_A1* | 0 | 0 | 0 | 0 | 0 | 0 | 0 | 13 | 2 | 0 | 15 |
| *7_A1* | 0 | 0 | 0 | 0 | 5 | 6 | 7 | 54 | 22 | 9 | 103 |
| *2_A1* | 2 | 0 | 26 | 123 | 39 | 65 | 13 | 58 | 40 | 3 | 369 |
| *6_A1* | 0 | 0 | 0 | 0 | 0 | 5 | 2 | 62 | 147 | 136 | 352 |
| *1_A1* | 0 | 0 | 0 | 0 | 2 | 10 | 0 | 89 | 40 | 3 | 144 |
| *8_A1* | 0 | 17 | 100 | 57 | 176 | 345 | 13 | 105 | 44 | 35 | 892 |
| *17_A2* | 0 | 0 | 0 | 0 | 0 | 0 | 0 | 8 | 0 | 0 | 8 |
| *16_A2* | 0 | 0 | 0 | 0 | 0 | 0 | 0 | 13 | 0 | 0 | 13 |
| *13_A2* | 0 | 0 | 0 | 0 | 0 | 0 | 9 | 371 | 1038 | 877 | 2295 |
| *10_A2* | 0 | 0 | 0 | 0 | 0 | 4 | 3 | 31 | 9 | 10 | 57 |
| *15_A2* | 0 | 0 | 0 | 0 | 0 | 2 | 0 | 0 | 0 | 0 | 2 |
| *22_A2* | 0 | 21 | 3 | 26 | 15 | 1 | 0 | 0 | 0 | 0 | 66 |
| *3_A2* | 0 | 0 | 0 | 0 | 4 | 7 | 13 | 33 | 30 | 0 | 87 |
| *24_A1 (US-1)* | 20 | 0 | 0 | 0 | 0 | 0 | 0 | 0 | 0 | 0 | 20 |
| *misc.** | 7 | 13 | 12 | 0 | 11 | 24 | 4 | 34 | 58 | 32 | 195 |
| Total | 34 | 85 | 188 | 264 | 290 | 581 | 73 | 899 | 1452 | 1118 | 4984 |

* misc. category corresponds to a group of *P. infestans* isolates from MLGs that occurred at a very low frequency in a single year.

**Table S4 Sampling details and multilocus genotypes (MLGs) of 28 *P. infestans* isolates derived from infected leaves collected in August 2004 in Lower Saxony (northern Germany) and Drenthe and Flevoland (northeast Netherlands).** Samples kindly provided by Bayer CropScience. The *13_A2* MLG was not observed in other regions of the Netherlands in 2004 or in previous years (Geert Kessel, PRI, Wageningen, The Netherlands, Personal Communication).

| **Collection date** | **Country** | **Region** | **Isolate name** | **MLG** |
| --- | --- | --- | --- | --- |
| 05/08/2004 | Germany | Lower Saxony | 04GE113 | *misc** |
| 05/08/2004 | Germany | Lower Saxony | 04GE114 | *13_A2* |
| 23/08/2004 | Germany | Lower Saxony | 04GE174 | *13_A2* |
| 23/08/2004 | Germany | Lower Saxony | 04GE193 | *2_A1* |
| 26/08/2004 | Netherland | Flevoland | 04NDL201 | *13_A2* |
| 30/08/2004 | Netherlands | Drenthe | 04NDL224 | *13_A2* |
| 30/08/2004 | Netherland | Drenthe | 04NDL219 | *13_A2* |
| 30/08/2004 | Netherlands | Drenthe | 04NDL220 | *13_A2* |
| 30/08/2004 | Netherlands | Drenthe | 04NDL238 | *13_A2* |
| 30/08/2004 | Netherlands | Drenthe | 04NDL223 | *13_A2* |
| 30/08/2004 | Netherlands | Drenthe | 04NDL229 | *13_A2* |
| 30/08/2004 | Netherland | Drenthe | 04NDL217 | *13_A2* |
| 30/08/2004 | Netherland | Drenthe | 04NDL218 | *13_A2* |
| 30/08/2004 | Netherlands | Drenthe | 04NDL228 | *13_A2* |
| 30/08/2004 | Netherlands | Drenthe | 04NDL236 | *13_A2* |
| 30/08/2004 | Netherlands | Drenthe | 04NDL226 | *13_A2* |
| 30/08/2004 | Netherlands | Drenthe | 04NDL221 | *13_A2* |
| 30/08/2004 | Netherlands | Drenthe | 04NDL222 | *13_A2* |
| 30/08/2004 | Netherlands | Drenthe | 04NDL239 | *13_A2* |
| 30/08/2004 | Netherlands | Drenthe | 04NDL241 | *13_A2* |
| 30/08/2004 | Netherlands | Drenthe | 04NDL235 | *13_A2* |
| 30/08/2004 | Netherlands | Drenthe | 04NDL230 | *2_A1* |
| 30/08/2004 | Netherlands | Drenthe | 04NDL240 | *13_A2* |
| 30/08/2004 | Netherlands | Drenthe | 04NDL227 | *13_A2* |
| 30/08/2004 | Netherlands | Drenthe | 04NDL231 | *13_A2* |
| 30/08/2004 | Netherlands | Drenthe | 04NDL232 | *13_A2* |
| 30/08/2004 | Netherlands | Drenthe | 04NDL233 | *13_A2* |
| 30/08/2004 | Netherlands | Drenthe | 04NDL234 | *13_A2* |

**misc*. category corresponds to a group of *P. infestans* isolates from MLGs that occurred at a very low frequency in a single year.

**Table S5 Details of the 26 *P. infestans* isolates used for the foliar aggressiveness studies.** Isolates labelled ^F^ were used for the mark-and-recapture field study. The full name of the isolates from 2006 are shown in the table below but elsewhere referred to using the shorter prefix of only 06. For each of the 26 isolates the multilocus genotype (MLG) is indicated.

| **Isolate name** | **Short code** | **Year of isolation** | **Country** | **Mating type** | **Cultivar** | **MLG** | **Virulence^e^** |
| --- | --- | --- | --- | --- | --- | --- | --- |
| 2006_3888A^F^ | *2_A1_1* | 2006 | GB | A1 | Lady Rosetta | 2_A1 | 1,3,4,7,8,10,11 |
| 2006_4068B | *2_A1_2* | 2006 | GB | A1 | Charlotte | 2_A1 | 1,3,4,7,8,10,11 |
| 2006_4100A^F^ | *6_A1_1* | 2006 | GB | A1 | Marfona | 6_A1 | 1,3,4,7,10,11 |
| 2006_3920A | *6_A1_2* | 2006 | GB | A1 | Estima | 6_A1 | 1,3,4,7,10,11 |
| 2006_4168B^F^ | *7_A1_2* | 2006 | GB | A1 | Charlotte | 7_A1 | 1,2,3,4,5,6,7,10,11 |
| 2006_4168C | *7_A1_1* | 2006 | GB | A1 | Charlotte | 7_A1 | 1,2,3,4,5,6,7,8,10,11 |
| 2006_4232E | *8_A1_2* | 2006 | GB | A1 | Marfona | 8_A1_2 | n/a |
| 2006_4256B^F^ | *8_A1_1* | 2006 | GB | A1 | Marfona | 8_A1_1 | 1,3,4,7,10,11 |
| 2006_3928A^F^ | *13_A2_3* | 2006 | GB | A2 | King Edward | 13_A2 | 1,2,3,4,5,6,7,8,9,10,11 |
| 2006_4132B | *13_A2_4* | 2006 | GB | A2 | Estima | 13_A2 | 1,2,3,4,5,6,7,8,9,10,11 |
| 2006_3964A | *13_A2_2* | 2006 | GB | A2 | King Edward | 13_A2 | 1,2,3,4,5,6,7,8,9,10,11 |
| 2006_3884B | *13_A2_1* | 2006 | GB | A2 | Maris Piper | 13_A2 | 1,2,3,4,5,6,7,8,9,10,11 |
| 2006_3936C2 | *10_A2_2* | 2006 | GB | A2 | Unknown | 10_A2 | 1,3,4,7,8,10,11 |
| 2006_4440C | *10_A2_1* | 2006 | GB | A2 | Maris Piper | 10_A2 | 1,3,4,7,8,10,11 |
| 2006_4012F | *3_A2_2* | 2006 | GB | A2 | Charlotte | 3_A2 | 1,2,3,4,6,7,10,11 |
| 2006_4244E | *2_A2_1* | 2006 | GB | A2 | Other Maincrop | 3_A2 | 1,2,3,4,5,6,7,8,10,11 |
| 2006_4388D | *17_A2_1* | 2006 | GB | A2 | Other Maincrop | 17_A2 | 1,2,3,4,5,6,7,8,10,11 |
| C2-95.17.3.2 | *8_A1_3* | 1995 | GB | A1 | Unknown | 8_A1_1 | n/a |
| C4-96.9.5.1 | *5_A1_1* | 1996 | GB | A1 | Unknown | 5_A1 | n/a |
| NL06269^a^ | *13_A2_NL1* | 2006 | NL | A2 | Unknown | 13_A2 | 1,2,3,4,5,6,7,8,9,10,11 |
| NL04246^a^ | *13_A2_NL2* | 2004 | NL | A2 | Unknown | 13_A2 | 1,2,3,4,5,6,7,8,9,10,11 |
| SE03058^b^ | *misc_SE_1* | 2003 | SE | A1 | Unknown | unique | 1,3,4,6,7,10,11 |
| SE03087^b^ | *misc_SE_2* | 2003 | SE | n/a | Unknown | unique | 1,3,4,7,8,10,11 |
| MP618^c^ | *misc_PL_2* | 2005 | PL | A2 | Unknown | unique | 1,3,4,6,7,10,11 |
| MP622^c^ | *misc_PL_1* | 2005 | PL | A1 | Unknown | unique | 1,3,4,6,7,10,11 |
| LD151^d^ | *8_A1_IRE* | n/a | Ireland | A1 | Unknown | 8_A1 | n/a |

Isolates kindly provided by ^a^Geert Kessel, Plant Research International, Wageningen, The Netherlands; ^b^Björn Andersson, Swedish University of Agricultural Sciences, Uppsala, Sweden; ^c^Renata Lebecka Plant Breeding and Acclimatization Institute - National Research Institute, Mlochow, Poland; ^d^Leslie Dowley, Teagasc, Oak Park, Carlow, Ireland. ^e^Results from a detached leaflet assay of virulence , the results of which differ slightly from the whole plant test results shown in Figure S13. In the case of MLG *13_A2* detached *R8* and *R9* leaflets are susceptible whereas whole plants represent a more rigorous test and score as resistant. Country abbreviations: GB, Great Britain; NL, The Netherlands; SE, Sweden; PL, Poland.

**Table S6 Potato cultivars selected for testing the aggressiveness of *P. infestans* genotypes and their foliar and tuber blight resistance ratings from the British Potato Council Cultivar database (July 2008).**

| **Potato cultivar** | **Foliar blight resistance** | **Tuber blight resistance** |
| --- | --- | --- |
| Lady Balfour | 8 | 7 |
| Cara | 7 | 7 |
| Estima | 4 | 5 |
| Maris Piper | 4 | 5 |
| King Edward | 3 | 4 |

**Table S7 Genome alignment statistics in the sequenced *13_A2* *P. infestans* 06_3928A isolate.** Pair-end reads of 06_3928A isolate were aligned to the reference genome strain T30-4 with BWA software package v0.5.7 .

| **Run** | **Lane** | **Read length (bp)** | **No. of reads (76bp X 2)** | **No. of mapped reads** | **% of mapped reads** | **No. of reads mapped in pairs** | **% of reads mapped in pairs** | **No. of unmapped reads** | **% of unmapped reads** |
| --- | --- | --- | --- | --- | --- | --- | --- | --- | --- |
| ID99 | Lane 5 | 76 | 25,308,382 | 24,630,707 | 97.3 | 24,156,004 | 95.4 | 677,675 | 2.7 |
| ID101 | Lane 8 | 76 | 27,448,558 | 26,432,076 | 96.3 | 25,770,274 | 93.9 | 1,016,482 | 3.7 |
| ID103 | Lane 5 | 76 | 35,037,640 | 33,174,380 | 94.7 | 31,971,342 | 91.2 | 1,863,259 | 5.3 |
| ID103 | Lane 6 | 76 | 34,627,312 | 32,693,366 | 94.4 | 31,527,118 | 91.0 | 1,933,946 | 5.6 |
| ID103 | Lane 7 | 76 | 35,689,613 | 33,930,316 | 95.1 | 32,770,200 | 91.8 | 1,759,296 | 4.9 |
| ID103 | Lane 8 | 76 | 33,410,173 | 31,938,448 | 95.6 | 30,956,230 | 92.7 | 1,471,725 | 4.4 |
|  |  | Total | 191,521,678 | 182,799,293 | 95.6 | 177,151,168 | 92.7 | 8,722,383 | 4.4 |
|  |  |  | Estimated genome depth | 58x |  |  |  |  |  |

**Table S8 Genome features of three sequenced *P. infestans* isolates.** The genome features described in the table were estimated from the alignment of Illumina reads of each isolate against the reference genome strain T30-4. The re-sequenced isolates PIC99189 and 90128 were previously reported .

| **Genome features** | ***P. infestans* 06_3928A** | ***P. infestans* isolates (clade1c)** | |
| --- | --- | --- | --- |
|  |  | **PIC99189** | **90128** |
| Predicted genome size (Mb) | 240 | - | **-** |
| Average genome coverage | 58x | - | **-** |
| Average breadth of coverage in coding sequences (%) | 99.2 | - | - |
| Average depth of coverage in coding sequences | 70.2x | - | - |
| No. of SNPs in coding sequences | 22,523 | 20,637 | 21,370 |
| SNP frequency per Kb in coding sequences | 1.008 | 0.940 | 1.031 |
| No. of SNPs causing loss of stop codons | 90 | 72 | 73 |
| No. of unique SNPs in coding genes* | 11,795 | 9,935 | 11,645 |
| No. of genes with at least one SNP* | 5,879 | 6,784 | 7,361 |
| No. of SNPs in introns | 6,043 | 4,673 | 4,658 |
| No. of SNPs in non-coding DNA | 155,996 | 76,738 | 97,078 |
| dS in T30-4 CDSs (syn. SNPs per syn. site) | 0.0018 | 0.0016 | 0.0016 |
| No. of genes with presence/absence (no reads) | 47 | 11 | 21 |
| Uncovered regions in the genome (no reads) (Mb) | 6.5 | 7.8 | 13.4 |
| No. of genes showing CNV>1 | 320 | 177 | 230 |
| No. of genes showing dN/dS>1^†^ | 288 | 232 | 270 |

* count of SNPs causing loss of stop codons were omitted

† dN/dS rates were calculated using Yang method .

**Table S14 List of candidate assembled RXLR effectors from unmapped reads of *P. infestans* *13_A2* 06_3928A isolate.** This list includes the protein sequences of the six assembled RXLR effectors from the 06_3928A isolate.

| **Pex ID** | **Protein length (aa)** | **HMM score** | **Signal peptide length (aa)** | **Full length** | **RXLR starts at amino acid position** | **RXLR-EER motif** | **Similarity in *P. infestans* T30-4** | **Amino acid sequence** |
| --- | --- | --- | --- | --- | --- | --- | --- | --- |
| Pex644 | 188 | 0.993 | 22 | Yes | 43 | RFLR-EER | PITG_22798, RxLRsng233 | MRRCYILIAIAVVLSGIASVVADSSQDKLMAVEGDQTTGTVNRFLRRDDELSAENTEERIVAGDIPLSARMINNIYKVEKRIVDPKLADELLEKPGLKTLKTHLDAALPYSERAKVFERWHADGVDPSSITKALKVHPAIAKKYNTVSTMYDLYVKSAAIKRLTELKRKSDNDLADAVRLKRQRINEZ |
| Pex50259 | 154 | 0.997 | 21 | No | 40 | RSLR-EER | NA | MHLRNALVWVVTTLLIGSVASDHPTVFQHFNGKVNALSSRSLRLHEERGIPVSTIANIKGMLTSKRVSDKTLDSWRKAGKTADKVFVWLSLGRGKGELFDNPNFAKWVKYVDDLSASHPERKSSISTLTSYYDDEPLSKMIIAAQKNPDTRALA |
| Pex30588 | 137 | 0.999 | 21 | No | 51 | RSLR-XXX | NA | MRRSSILYVAAVALCISFCDAASAATNSEFSPIMPFGTLQSAYSTALTSTRSLRGSKRDDDNKDMDFVQENRAGIQLTHIDDLLKQLALNEKMVLQNLNKFDDDLMRKLRQNPSWARTILRWKDRDLHPTQVAAILN |
| Pex46622 | 126 | 0.999 | 20 | Yes | 41 | RLLR-EER | (PITG_09739, PITG_09773) RxLRfam6 | MRISQAVVVVTVAFLASSEALSTRMDDKVSKVATHDGPSQRLLRIHHTAIEDEDDSEERGLKEKDFKRLAVYADELGINVEKATKNTAYLREVADEYAKYKSLLNQLIKKRKSKGSPMITYEHHGZ |
| Pex15083* | 117 | 0.977 | 20 | Yes | 49 | RLLR-EER | (PITG_22870, PITG_08943) *Avr2*, RxLRfam7 | MRLAYIFAVTMAGALPYCNALHAAPGAKALNKIKTFPDFAAPSRMDGNRLLRRVDNEESETEEERGFNLKDTLKKLNPIKAAGKAKDKAKEVTEKITDADWKKLVNYLQSKGNKRSZ |
| Pex14182 | 111 | 0.998 | 21 | Yes | 43 | RFLR-EER | NA | MRGVETILTAVLCILCGTTDAAMTSDETIAASVATKNGVLAKRFLRAQGPPDEERGRLKDVFEKVKRLARYNKWIFSDKSPDWVDKKYPQFSQGYEKFWENRLVGGGKYAZ |

*Pex15083 assembled RXLR effector was reported as *Avr2-like*, a variant of the *P. infestans* avirulence *Avr2* gene present in the isolate 06_3928A. *Avr2-like* is not recognized by the cognate plant immune receptor *R2* . The abbreviation ‘aa’ correspond to amino acids.

**Table S15 Validation of candidate assembled RXLR effectors from unmapped reads of *P. infestans* *13_A2* isolate 06_3928A according to the PCR assay.** *P. infestans* isolates from *13_A2* genotype are highlighted in blue. A plus indicates that the gene was amplified and a minus that the gene could not be amplified.

| ***P. infestans* strain** | **Multilocus**  **genotype**  **(MLG)** | **PCR product amplification for** | | | | | |
| --- | --- | --- | --- | --- | --- | --- | --- |
|  |  | ***Pex644*** | ***Pex50259*** | ***Pex30588*** | ***Pex46622*** | ***Pex15083*** | ***Pex14182*** |
| T30-4 | ***misc**** | **-** | - | - | **-** | - | - |
| 2006_3928A | ***13_A2*** | **+** | **+** | **+** | **+** | **+** | **+** |
| 2006_3884B | *13_A2* | + | + | + | + | + | + |
| 2006_3964A | *13_A2* | + | + | + | + | + | + |
| 2006_4132B | *13_A2* | + | + | - | + | + | + |
| 2006_4012F | *3_A2* | - | + | - | + | + | - |
| 2006_4244E | *3b_A2* | - | + | - | + | + | + |
| 2006_3936C2 | *10_A2* | - | + | - | + | - | + |
| 2006_4440C | *10_A2* | - | + | - | + | - | + |
| 2004_7804B | *15_A2* | - | - | - | - | - | + |
| 2006_3992G | *16_A2* | + | + | + | + | + | - |
| 2006_4388E | *17_A2* | - | + | - | - | + | + |
| 2003_25_1_3 | *22_A2* | - | + | + | - | + | + |
| 2003_25_3_1 | *22_A2* | + | + | + | - | + | + |
| 2006_3984C | *1_A1* | + | + | + | - | + | + |
| 2006_4304A | *1_A1* | + | + | + | - | + | + |
| 2006_3888A | *2_A1* | + | + | + | + | + | + |
| 2006_4068B | *2_A1* | + | + | + | + | + | + |
| 2006_3960A | *2_A1* | - | + | + | + | + | + |
| 2006_4352E | *4_A1* | + | - | + | + | - | + |
| 1996_9_5_1_C4 | *5_A1* | - | - | + | - | + | + |
| 07_5866C | *5g_A1* | - | - | + | + | + | + |
| 2006_3920A | *6_A1* | + | + | - | + | - | + |
| 2006_4100A | *6_A1* | + | + | - | + | - | + |
| 2006_4168B | *7_A1* | + | - | - | - | + | + |
| 2006_4168C | *7_A1* | + | - | - | - | + | + |
| 2006_4232E | *8_2a_A1* | + | - | - | - | + | + |
| 2006_4256B | *8a_A1* | + | - | - | - | + | + |
| 2006_4320F | *12_A1* | + | - | - | - | + | + |

**misc*. category in this case refers to the fact that T30-4 is a product of a laboratory cross and does not fit into the clonal population structure.
