# Supplementary material for: Genome Analyses of an Aggressive and Invasive Lineage of the Irish Potato Famine Pathogen
Source: PLoS Pathog. 2012 Oct 4;8(10):e1002940. doi: 10.1371/journal.ppat.1002940 (PMC3464212; doi:10.1371/journal.ppat.1002940)
Supplement: Text S3 — Supplementary Materials and Methods; (S3a) Pathogen sampling and genotyping: (S3b) Aggressiveness and virulence testing: (S3c) Pathogen whole-genome and expression analyses. (DOC) [file ppat.1002940.s003.doc]

**Text S3a Population sampling and pathogen genotyping**

***i) Population sampling***

The *P. infestans* isolates used in this analysis were sampled as follows. Over the course of 2003, 2004 and 2005 seasons across Great Britain (GB), a single lesion sample per reported blight outbreak (*n*=340 outbreaks) was supplied by industry blight scouts as part of a Potato Council monitoring programme. Each lesion was sent to The Food and Environment Research Agency (Fera, York, UK) before passing to The James Hutton Institute (JHI) within a cut potato tuber. At JHI the pathogen was isolated on rye A agar with antibiotics (chloramphenicol 34 µg/ml, rifampicin 30 µg/ml, ampicillin 150 µg/ml, pimaricin 10 µg/ml) and mycelium was produced for DNA extraction by growing on clear pea broth (125g frozen peas, 1L distilled water). Almost all samples were obtained from infected leaves or stems of commercial potato crops with a small proportion from gardens, allotments or other hosts such as solanaceous weeds or tomatoes. Over the 2003 and 2004 seasons more detailed sampling of commercial potato crops and home gardens in Scotland was conducted by staff at the JHI with up to 15 lesions sampled per disease outbreak (*n*=116 outbreaks). Over the 2006, 2007 and 2008 seasons from across GB, infected crops were identified by Potato Council blight scouts, as above, but 8 or more samples were provided per blight outbreak (*n*=672 outbreaks). The locations of the sampled 2006-8 outbreaks are shown (Figure S1 in Text S1). In previous GB surveys 3,145 isolates (*n*= 436 outbreaks) were collected from which a sample of 537 isolates from a wide geographical range was selected for SSR analysis in this study.

***ii) Pathogen genetic characterisation***

The multilocus genotype (MLG) *13_A2* was first examined by RFLP fingerprinting using the RG57 probe . Seven isolates collected from Great Britain (GB) in 2005 were defined according to a standard notation and shown to differ from A2 lineages previously seen in GB (Table S2A in Text S2). The mtDNA haplotype was defined as *Ia* and, consistent with another study , all isolates were shown to be fully resistant to the fungicide metalaxyl . All subsequent genotyping was based on SSRs. DNA was extracted from freeze-dried samples of mycelium from each isolate and typed using 11 SSR loci (protocol on [www.eucablight.org](http://www.eucablight.org/)). The SSR peaks were scored in GeneMapper 3.7 (Applied Biosystems) and processed in an MS Excel spreadsheet to define MLGs which were named with a number followed by the mating type of isolates in that MLG (Table S2B in Text S2). The markers proved to have a resolution appropriate for discrimination of MLGs with, at most loci, a stable SSR profile amongst many hundreds of isolates collected over several years (Table S2 in Text S2). Consistent minor variation in one or two highly variable SSR loci (primarily G11 and D13 (Figure S2 in Text S1) differentiated sub-types within specific MLGs (Figure 1B and Table S2B in Text S2). Such variation has been reported within *P. ramorum* clonal lineages and these data on *P. infestans* will be reported in more detail in subsequent publications. Isolates with novel combinations of alleles across several loci and found at a very low frequency and commonly in only a single blight outbreak were grouped into a ‘miscellaneous’ MLG category termed ‘*misc’*. The overall numbers and frequencies of each MLG in each of the seasons are presented (Figure 1 and Table S3 in Text S2). In some MLGs three alleles were consistently observed which is suggestive of differences in ploidy. Such variation in ploidy at a local or whole genome level has been reported previously in *P. infestans* with a range of methods .

**Text S3b Aggressiveness and virulence testing**

***i) Isolate selection***

Isolates of *P. infestans* were selected as follows; four isolates of multilocus genotype (MLG) *13_A2*, pairs of isolates representing other dominant MLGs from the 2006 Great Britain (GB) population, additional isolates from other parts of Europe (The Netherlands, Sweden and Poland) and two reference isolates from Scotland in the 1990s. The isolates from the Netherlands were MLG *13_A2* whereas isolates from SE and PL had SSR MLGs distinct from all those observed in the GB samples (Table S5 in Text S2).

***ii) Laboratory studies of foliar aggressiveness***

Aggressiveness was measured in an experiment that was carefully controlled for environmental conditions, duration the isolate has been in axenic culture, whether the isolate has been ‘passaged’ on leaf material prior to testing, the potato varietal resistance and the developmental stage of the potato leaves tested. The aggressiveness of *P. infestans* isolates is likely to be affected by the length of time in axenic culture and it is thus important to ‘passage’ isolates on leaf material to restore aggressiveness prior to any experiment. Isolates were thus inoculated onto detached leaflets of glasshouse-grown potato cultivar Craig’s Royal (which has no known *R* genes) in individual sealed boxes lined with damp tissue paper. Sporangia were harvested after one week and used to re-inoculate more leaves of the same cultivar. Each isolate was ‘passaged’ by this method at least three times prior to use. Five contemporary potato varieties were selected with a range of blight resistances (Table S6 in Text S2). Three tubers of each potato cultivar per pot were planted in compost in 30 cm pots and grown in the glasshouse for 5-6 weeks. For each cultivar, leaves of a similar age and size (i.e. avoiding the youngest and oldest leaves) were harvested and individual leaflets placed in clear plastic boxes (26 leaflets per box) lined with moist tissue paper. Sporangial inoculum of each of the 26 isolates was washed off the infected Craig’s Royal leaflets and adjusted to 14,000 sporangia per ml before cooling at 4^o^C to encourage zoospore release. A droplet of 30 μl of inoculum from each isolate (approx 420 sporangia) was applied to the centre of each leaflet. A total of 60 boxes of leaves were inoculated and 30 placed in a randomised block design with six replicate blocks in each of two adjoining illuminated walk-in growth rooms set at a constant 18^o^C or 13^o^C with 16/8 hours of light and dark. The 1,560 leaves were scored daily for first symptoms (i.e. infection period, IP), and sporulation (i.e. latent period, LP) and at 6 days post inoculation (dpi) lesion size was measured in two orientations at right angles to each other using electronic callipers. A separate study comparing the aggressiveness of strains 06_3928A and T30-4 was conducted on King Edward and Maris Piper using the same methods and environmental conditions. Analysis of variance was conducted on the 13^o^C and 18^o^C data separately using GenStat for Windows (14^th^ Edition).

***iii) Field test of aggressiveness - ‘mark and recapture’ trial***

A randomised complete block (four blocks) field trial design comprising four 25-plant plots of each of five cultivars (Table S6 in Text S2) was planted on 9 May 2007. Each plot was laid out as a 5 x 5 plant grid with a surrounding ‘guard row’. Once the plants had met in the rows the trial was sprayed once a week with Bravo (chlorothalonil) until 10 July to minimise the risk of blight entering the trial from local crops. Inoculum of a MLG *13_A2* isolate and four other MLGs (Table S5 in Text S2) was prepared as described above and all five isolates mixed in equal proportions. Approximately 50 ml of the mixed zoospore suspension was used to spray-inoculate the lower leaves of the central plant (plant 13) of each plot late in the afternoon of 18^th^ July 2007 when rain was forecast. As a control of the inoculum quality, each zoospore suspension was used to inoculate detached leaves in the laboratory and all became infected. From 18^th^ July onwards thirty minutes of mist irrigation was applied morning and afternoon over the whole trial to encourage infection and disease spread. By 24^th^ July blight symptoms were observed in the central plant of most plots and by the 30^th^ July disease had spread to the plants adjacent to plant 13 and the sampling of leaves with clear single blight lesions began. Since the objective was to examine secondary spread of blight plant 13 was not sampled. Sample numbers varied according to the stage of the epidemic but the target was to sample 10 blight infected leaves per plot on each of five dates (*n*= 1,000 lesions) up until 20^th^ August. Leaves with single lesions, primarily from the eight plants adjacent to the central plant, were selected and placed in individual plastic bags to prevent cross contamination. In the laboratory a section of each lesion margin was pressed onto an FTA card (Cat. No. WB120205, Whatman, UK) using a pair of pliers and the cards were air dried and stored. Small (2mm) disks were cut out from the cards and processed according to the manufacturer’s instructions (Whatman FTA plant Protocol BD05) prior to placing into SSR genotyping PCR mixes (see protocol on [www.eucablight.org](http://www.eucablight.org/)). SSR profiles for 716 lesions (36 lesions per plot) were determined and the fingerprints compared to those of the MLGs initially released into the trial. Clear foci of disease were noted around the centre of each plot and, irrespective of cultivar, MLG *13_A2* was responsible for almost all blight lesions in the trial throughout the epidemic (Figure 3). Only 35 of 716 lesions were of an MLG other than *13_A2*. No association between *P. infestans* MLG and cultivar was observed.

***iv) Virulence and host resistance testing***

The virulence testing and host resistance screening of isolates against Black’s differential series was carried out on whole plants (Figure S13 in Text S1) or detached leaves (Table S5 in Text S2) according to published methods .

Virulence testing of isolates was carried out on stable transformant potato cv. Desiree plants carrying the immune receptor *R* genes *Rpi-blb1*, *Rpi-blb2* and *Rpi-vnt1.1*, respectively and compared to wild-type potato cv. Desiree. Detached leaf infection assays were conducted as follows. Sporangia of *P. infestans* strains 06_3928A and T30-4 were harvested in cold sterile distilled water after growing for 10-12 days on rye sucrose agar (RSA) plates. Sporangial solution was incubated for 3-4 hours to promote the release of zoospores. Detached potato leaflets were then drop-inoculated with 100,000 zoospores/ml of *P. infestans* 06_3928A or T30-4. Infected lesions were scored at 5 days post inoculation (dpi). Wild-type Desiree potato plants were also infected with *P. infestans* strains 06_3928A and T30-4 and used as a positive control (Figure 8B).

**Text S3c Pathogen whole-genome and expression analyses**

***i) Library preparation and sequencing***

For the genomic DNA extraction, *Phytophthora infestans* isolate 06_3928A was cultured in Rye Sucrose Agar (RSA) plates at 18°C for 12 days. Plugs with mycelium of *P. infestans* isolate 06_3928A were transferred to modified plich medium , grown for another two weeks at 18°C and then harvested for genomic DNA isolation using Omniprep© kit (GBiosciences, Cat No. 786-136) with minor modifications. For sequencing, the flow cells were prepared according to the manufacturer's instructions using Illumina pair read cluster generation kit PE-203-4001. Sequencing reactions were performed mostly on 2G GAs (Illumina Inc.). The reference genome sequence of *P. infestans* strain T30-4, annotation and gene/exon locations was downloaded from www.broad.mit.edu (GenBank project accession number AATU01000000).

***ii) Alignment of reads to the reference genome***

The generated Illumina raw reads from 06_3928A having abnormal lengths and reads containing Ns were removed from the datasets using in-house scripts. Filtered Illumina reads were used to align to the reference genome *P. infestans* strain T30-4 with Burrows-Wheeler Transform Alignment (BWA) software package v0.5.7 with a seed length (l) of 38 and a maximum of mismatches (M) allowed of 3 as parameters. In total, 95.6% of the Illumina reads from 06_3928A aligned to the T30-4 genome (Table S7 in Text S2).

***iii) De novo assembly of unmapped reads***

We extracted 8,722,383 unmapped reads of the isolate 06_3928A from genome alignments in SAM format using “SAMTOOLS view flag 4” option of SAMTOOLS software package v0.1.7 (http://samtools.sourceforge.net/) and in-house scripts. Unmapped reads were assembled using the VELVET software package v1.0.18 with a Kmer of 53, a minimum contig length of 200 bp nucleotides and an insertion length of 300 bp as parameters. We obtained 15,654 contigs with a N50 of 359 bp, a mean size of 367 bp and a median size of 278 bp. The smallest contig size was 201 bp and the largest contig size 6,286 bp. The obtained contigs are equivalent to 5.4 Mb in size. Then, all 15,654 contigs were mapped back to the reference genome strain T30-4 using NUCmer program, included in MUMmer software package v3.2 . A total of 9,838 contigs that are equivalent to 2.77 Mb of the assembly showed hits to T30-4 and were filtered out of the assembly. The remaining 5,816 contigs were kept for the next steps of the analysis of the genes encoding proteins, which included prediction of secretion signals and RXLR motifs.

***iv) Prediction of secreted proteins and RXLR motif from assembled contigs***

The authors wrote a script to search for putative open reading frames (ORFs) in 5,816 assembled contigs (containing coding and non coding DNA). We identified coding sequences with putative start codons which were translated to amino acids using another script written by the authors. Signal peptides were predicted from predicted proteins using SignalP v2.0 and TMHMM programs. Secreted proteins were selected when showing a SignalP HMM score cut-off of > 0.9 and NN cleavage site within 10 and 40 amino acids. Secreted proteins were predicted to contain RXLR motifs when: RXLR position was present within 30 and 60 amino acids, RXLR position was higher than NN cleavage site and signal peptide length was <= 30 . The RXLR prediction resulted in the identification of six candidate RXLR effectors in the isolate 06_3928A (Table S14 in Text S2). Six RXLR effectors predicted with this pipeline were validated by PCR (see Text S3e)

***v) PCR validation of candidate assembled RXLR genes***

Assembled RXLRs were validated by PCR amplification of genomic DNA from a collection of 29 isolates of many MLGs in a 20 μl reaction volume using a Primus 96^plus^ Thermalcycler (MWG-Biotech, Ebersberg, Germany). We used specific primers for the amplification of six candidate assembled RXLRs genes: *Pex644, Pex50259, Pex30588, Pex46622, Pex15083* and *Pex14182* with an expected size of 514, 258, 257, 365, 472 and 859 bp respectively (*Pex644*_F: TGAGTGGAATCGCATCAGTAGT, *Pex644*_R: ATCCTCTGCCTTTTTAATCTGAC, *Pex50259*_F: TGGCAAGGTAAACGCTCTCT, *Pex50259*_R: GAGGCCGATAAGTCGTCAAC, *Pex30588*_F: TTTCTGTGATGCTGCCTCTG, *Pex30588*_R: CGTCAAACTTGTTAAGGTTTTGC, *Pex46622*_F: ATGCGTATCTCGCAAGCT, *Pex46622*_R: TCATACGTGATCATCGGAGA, *Pex15083*_F: ACGCTTCTATCCGACAACGA, *Pex15083*_R: ATTGGTGGTAATGCCTGCG, *Pex14182*_F: ATGCGTGGCGTCGAAACTA, *Pex14182*_R: CCATTGGCTGATACGGTATTT). Each reaction contained 1 × GoTaq*®* Flexi buffer, 20 μg BSA, 1.5 mM MgCl_2_, 100 μM dNTPs, 0.8 unit of Taq polymerase (GoTaq*®* DNA polymerase, Promega), 0.2 μM of primers and 20 ng of template DNA. Amplification conditions consisted of one cycle of 94°C for 4 min, 30 cycles of 94°C for 20 s, 60°C for 20 s, 72°C for 45 s and a final cycle of 72°C for 5 min. All six assembled RXLR genes (*Pex644, Pex50259, Pex30588, Pex46622, Pex15083* and *Pex14182*) evaluated by PCR were found to be present in 06_3928A and absent in the reference genome strain T30-4 (Table S15 in Text S2).

***vi) Optimization of SNP calling parameters***

A False Discovery Rate (FDR) analysis was used to determine the performances for single nucleotide polymorphism (SNP) calling in 90% identical genome regions of *P. infestans* 06_3928A genome. FDR for the SNP calling methods were calculated by randomly introducing 100,000 SNPs into the coding sequences of the 90% identical genome regions of 06_3928A genome, aligning re-sequenced 06_3928A reads to the ‘modified’ reference. A total of 54 parameter sets (Figure S14 in Text S1) were tested as a function of (i) a minimum read depth of coverage at SNP position, artificially limited to 3, 4, 5, 6, 7, 8, 9, 10, 12, 14, 16, 18, 20, 22, 24, 26, 28, 30 and (ii) percentage of reads specify a SNP, also artificially limited to (80, 90, 95). For FDR analysis we measured: accuracy, specificity and sensitivity. Accuracy was defined as (TP + TN)/(TP + FP + FN + TN), specificity as TN/(TN + FP) and sensitivity as TP/(TP + FN) where TP is the number of true positives, TN is the number of true negatives, FP is the number of false positives and FN is the number of false negatives. We found that 90% consensus among reads calling a SNP with a minimum of 10x coverage gave high specificity without reducing the sensitivity and accuracy. Also this parameter produces a low false positive rate (0.012%) and a high true positive rate (85.82%). Therefore this parameter was selected for the following analyses (arrow in Figure S14A in Text S1 points to the false positive and true positive rate of the selected parameter, and dashed line in Figure S14B in Text S1 correspond to the specificity, sensitivity and accuracy values of the selected parameter). Using this parameter we identified a total of 22,523 SNPs were detected in 06_3928A isolate (Table S8 in Text S2).

***vii) Breadth of coverage and presence/absence polymorphisms***

Breadth of coverage was calculated for each of the 18,155 genes as the percentage of nucleotides with at least one read aligned as previously described . Genes were considered absent conservatively when breadth equals 0. Genes were called absent when breadth of coverage equalled 0 in re-sequenced isolate 06_3928A (Table S8 in Text S2). We also discovered the absence of two genes previously described as contaminants in the reference genome strain T30-4 (PITG_23200 and PITG_23237) . Excluding the absences of the two contaminants, in total we predicted 47 coding genes missing in 06_3928A genome (Table S8 in Text S2 and Table S13).

***viii) Estimation of copy number from average read depth***

Average Read Depth for the CDS of a gene ‘g’ ARD(g) was calculated and adjusted using GC content in a similar manner to published methods . Adjusted ARD for a gene AARD(g) belonging the ith GC content percentile was obtained by the formula AARD(g)= ARD(g) /mARDGC where mARDGC is the mean depth for genes in the ith GC content percentile. Copy Number for a gene ‘g’ CN(g) was calculated as AARD(g).mARD. Copy Number Variation for a gene ‘g’ in a strain ‘s’ is then given by: CNV(g,s) = [CN(g,s) /CN(g,T30-4)]-1; where CN(g,s) is the estimated copy number of ‘g’ in a strain ‘s’. As a result, an absent gene will have a CNV value of -1, a single copy gene a CNV value of 0, a two-copy gene a CNV value of 1 and so on. The number of coding genes having at least one additional gene copy (CNV>1) in 06_3928A genome is listed in Table S8 in Text S2 and a more detailed annotation in Table S12.

***ix) PCR validation of RXLR genes showing with CNV events***

Deletion events in the *Avr1* gene (PITG_16663) were studied in PCR reactions carried out from genomic DNA in a 20 μl reaction volume using a Primus 96^plus^ Thermalcycler (MWG-Biotech, Ebersberg, Germany). We used specific primers for *Avr1* amplification with an expected size 506 bp (*Avr1*-F1: TTGCCCTGTTGTGTATGCA, and *Avr1*-R1 GGCCATCAATTCCTCAGGT). Each reaction contained 1 × GoTaq*®* Flexi buffer, 20 μg BSA, 1.5 mM MgCl_2_, 100 μM dNTPs, 0.8 unit of Taq polymerase (GoTaq*®* DNA polymerase, Promega), 0.2 μM of primers and 20 ng of template DNA. Amplification conditions consisted of one cycle of 94°C for 4 min, 30 cycles of 94°C for 20 s, 60°C for 20 s, 72°C for 45 s and a final cycle of 72°C for 5 min.

The duplication event in PITG_14787 and PITG_14783 was determined using real time ∆∆Ct method for q-PCR against the endogenous control gene *Actin* *A* gene *(ActA)* (GenBank M59715), assuming T30-4 to have a single copy. We used specific primers for the amplification of *ActA* and the secreted RXLR effectors PITG_14787 and PITG_14783 (*ACTA*-F2: CATCAAGGAGAAGCTGACGTACA, *ACTA*-R2: GACGACTCGGCGGCAG, PITG_14787-F: CCAAATGAGCAGCACCAACGTCAA and PITG_14787-R ACATCGTCAGCAATGCCTAAGTGC). With the exception of a single base change, that did not form part of the fragment amplified by the above primers, PITG_14787 and PITG_14783 were identical in DNA sequence. The above primers thus amplify both genes. Reactions were carried out in 25 µl reaction volume using a Chromo4™ Real-Time PCR Detector (Bio-Rad Life Sciences), based on Quantitect SYBR Green PCR Kit (Qiagen), with the following primer final concentrations *ActA*-F2 300 nM, *ActA*-R2 300 nM and PITG_14787-F 900 nM, PITG_14787-R 900 nM. DNA amplification conditions consisted of one cycle of 95°C for 15 min followed by 40 cycles of 95°C 15 s, 59°C 30 s and 72°C 30 s.

***x) Microarray analyses***

Mycelia were harvested after growing for 12 days in V8 juice Agar or Rye Sucrose Agar (RSA), ground in liquid nitrogen and frozen prior total RNA extraction. In addition to mycelia, we also prepared total RNA from infected potato material. We first collected sporangia in cold distilled sterile water and then incubated for 3-4 h at 4°C. After zoospores were released, we used 10 µl aliquot at a concentration of 100,000 zoospores/ml for drop inoculation on potato cultivar Desiree detached leaves and incubated at 18°C. We carried out infections by drop inoculation with zoospores of *P. infestans* 06_3928A and NL07434 isolates. We collected leaf discs of infected potato leaves of each isolate at 2, 3 and 4 days post inoculation (dpi). The infected material was ground in liquid nitrogen to a fine powder and frozen prior to total RNA extraction. Each sample and its biological replicate were homogenized with RLT buffer containing ß-mercaptoethanol from the RNeasy Plant Mini Kit (Qiagen, Cat No. 74904) proceeding with a modified manufacture’s protocol. RNA quality and integrity were checked prior to cDNA synthesis using the Bioanalyzer (Agilent 2100). NimbleGen microarray services were utilized for cDNA preparations and subsequent chip hybridizations to a custom array design (080603_PI_BH_EXP) that include all predicted genes in *P. infestans* and tomato ESTs. Microarray normalization was done using the previously described methods . Analysis of gene expression was performed using the MultiExperiment viewer (MeV). Log2 transformed array intensity values were analyzed for differential gene expression using the t-test, as implemented in MeV assuming equal variances. For this study, only the array targets corresponding to annotated *P. infestans* genes were analyzed. To identify genes that are induced *in planta*, T-tests were performed comparing sample replicates for mycelia grown in RSA and V8 to replicates of each time point post inoculation. Genes were considered differentially regulated when p <0.05. To test for the probability of calling a gene differentially regulated due to natural variability between sample replicates; we performed a False Discovery Rate (FDR) analysis as previously reported in the reference genome strain T30-4 . Differentially regulated genes at 2 and/or 3 dpi with p-value <0.05 and FDR probability <5% (q-value <0.05) were reported as significant. Significant *in planta*-induced genes exhibiting at least two-fold gene induction between averaged media and infected potato sample replicates (at 2 and/or 3 dpi) were considered induced during infection. In total, we detected 4,934 genes induced during infection in potato (at 2 and/or 3 dpi) in at least one of the three strains (Table S16).

***xi) Comparison of Cdc14 gene expression in T30-4 and 06_3928A during* *potato leaf infection***

Quantitative gene expression analysis of RXLR genes was performed as previously described . In brief, total RNA was extracted from samples (potato Desiree leaves drop-inoculated with T30-4 and 06_3928A at 1 to 5 days post inoculation (dpi) and incubated at 18^o^C) using RNeasy Plant RNA extraction kit (Qiagen, UK), DNAase treated with TURBO DNA-free (Ambion, UK) and cDNA was synthesized using an oligo dT primer using Superscript II® (Invitrogen, UK). Quantitative RT-PCR was performed with Power SYBR® Green (Applied Biosystems, UK) with a Chromo4® Real-Time Detector (Bio-rad, UK). ActinA was used as an endogenous control gene using the primers For 5’-CATCAAGGAGCTGACGTACA-3’ and Rev 5’-GACGACTCGGCGGCAG-3’. *Cdc14* expression was quantified using For 5’-TGCACTTTTAACTTGACTATTCTTGA-3’ and Rev 5’-AGATCAAACGTCTTAGTGGAGATG-3’. Data is presented as fold change relative to normalized expression at day 1 of each individual *P. infestans* isolate calculated by the ΔΔCt method with error bars representing +/- s.e.

***xii) Measurement of biotrophic growth during infection***

*P. infestans* strains T30-4, 06_3928A and NL07434 were grown in Rye Sucrose Agar (RSA) plates for 12 days at 18°C. Sporangia were harvested from RSA plates by adding cold water to the plates and zoospores were collected after one hour of incubation at 4°C. Potato leaves (cultivar Desiree) were inoculated with droplets of a solution of 100,000 zoospores/ml applied onto abaxial sides of detached leaflets on wet paper towels. Two droplets per leaflet were applied for each of the three isolates. We used a total of in 14 leaflets that correspond to 28 replicates per time point for each strain. Whole leaves UV digital images were recorded with GELDOC imaging system (Biorad) at 2, 3 and 4 days post inoculation (dpi). UV light exposed digitalized images were loaded in Image J (1.43u) software package to measure the biotrophic growth during lesion expansion across 2, 3 and 4 dpi. Using the area function of Image J we calculated the diameters for the outer (include both non-necrotic and necrotic region) and the inner ring (necrotic region only). Then we calculated the difference between the outer and the inner ring diameters to estimate the extent of the biotrophic growth (non-necrotic region). For each time point we estimated the standard error according to measurement of 28 replicates.

**REFERENCES IN SUPPORTING INFORMATION**

1. Caten CE, Jinks JL (1968) Spontaneous variability of single isolates of *Phytophthora infestans* .I. Cultural variation. Canadian Journal of Botany 46: 329-&.

2. Cooke DEL, Young V, Birch PRJ, Toth R, Gourlay F, et al. (2003) Phenotypic and genotypic diversity of *Phytophthora infestans* populations in Scotland (1995–97). Plant Pathology 52: 181-192.

3. Day JP, Wattier RAM, Shaw DS, Shattock RC (2004) Phenotypic and genotypic diversity in *Phytophthora infestans* on potato in Great Britain, 1995–98. Plant Pathology 53: 303-315.

4. Goodwin SB, Drenth A, Fry WE (1992) Cloning and genetic analyses of two highly polymorphic, moderately repetitive nuclear DNAs from *Phytophthora infestans*. Current Genetics 22: 107-115.

5. Forbes GA, Goodwin SB, Drenth A, Oyarzun P, Ordonez ME, et al. (1998) A global marker database for *Phytophthora infestans*. Plant Disease 82: 811-818.

6. Shaw DS, Nagy ZA, Evans D, Deahl K (2007) The 2005 population of *Phytophthora infestans* in Great Britain: the frequency of A2 mating type has increased and new molecular genotypes have been detected. PPO-Special Report No. 12: 137-143.

7. Griffith GW, Shaw DS (1998) Polymorphisms in *Phytophthora infestans*: four mitochondrial haplotypes are detected after PCR amplification of DNA from pure cultures or from host lesions. Appl Environ Microbiol 64: 4007-4014.

8. Gisi U, Walder F, Resheat-Eini Z, Edel D, Sierotzki H (2011) Changes of genotype, sensitivity and aggressiveness in *Phytophthora infestans* Isolates collected in European Countries in 1997, 2006 and 2007. Journal of Phytopathology 159: 223-232.

9. Lees AK, Wattier R, Shaw DS, Sullivan L, Williams NA, et al. (2006) Novel microsatellite markers for the analysis of *Phytophthora infestans* populations. Plant Pathology 55: 311-319.

10. Goss EM, Larsen M, Chastagner GA, Givens DR, Grunwald NJ (2009) Population genetic analysis infers migration pathways of *Phytophthora ramorum* in US nurseries. PLoS Pathog 5: e1000583.

11. Whittaker SL, Shattock RC, Shaw DS (1991) Variation in DNA content of nuclei of *Phytophthora infestans* as measured by a microfluorimetric method using the fluorochrome DAPI. Mycological Research 95: 602-610.

12. van der Lee T, Testa A, Robold A, van 't Klooster J, Govers F (2004) High-density genetic linkage maps of *Phytophthora infestans* reveal trisomic progeny and chromosomal rearrangements. Genetics 167: 1643-1661.

13. Stewart H, Bradshaw J, Wastie R (1994) Correlation between resistance to late blight in foliage and tubers in potato clones from parents of contrasting resistance. Potato Research 37: 429-434.

14. Kamoun S, Klucher KM, Coffey MD, Tyler BM (1993) A gene encoding a host-specific elicitor protein of *Phytophthora parasitica*. Molecular Plant-Microbe Interactions 6: 573-581.

15. Haas BJ, Kamoun S, Zody MC, Jiang RH, Handsaker RE, et al. (2009) Genome sequence and analysis of the Irish potato famine pathogen *Phytophthora infestans*. Nature 461: 393-398.

16. Li H, Durbin R (2009) Fast and accurate short read alignment with Burrows-Wheeler transform. Bioinformatics 25: 1754-1760.

17. Zerbino DR, Birney E (2008) Velvet: algorithms for de novo short read assembly using de Bruijn graphs. Genome Res 18: 821-829.

18. Kurtz S, Phillippy A, Delcher AL, Smoot M, Shumway M, et al. (2004) Versatile and open software for comparing large genomes. Genome Biol 5: R12.

19. Nielsen H, Engelbrecht J, Brunak S, von Heijne G (1997) A neural network method for identification of prokaryotic and eukaryotic signal peptides and prediction of their cleavage sites. Int J Neural Syst 8: 581-599.

20. Krogh A, Larsson B, von Heijne G, Sonnhammer EL (2001) Predicting transmembrane protein topology with a hidden Markov model: application to complete genomes. J Mol Biol 305: 567-580.

21. Torto TA, Li S, Styer A, Huitema E, Testa A, et al. (2003) EST mining and functional expression assays identify extracellular effector proteins from the plant pathogen *Phytophthora*. Genome Res 13: 1675-1685.

22. Win J, Morgan W, Bos J, Krasileva KV, Cano LM, et al. (2007) Adaptive evolution has targeted the C-terminal domain of the RXLR effectors of plant pathogenic oomycetes. Plant Cell 19: 2349-2369.

23. Raffaele S, Farrer RA, Cano LM, Studholme DJ, MacLean D, et al. (2010) Genome evolution following host jumps in the Irish potato famine pathogen lineage. Science 330: 1540-1543.

24. Yoon S, Xuan Z, Makarov V, Ye K, Sebat J (2009) Sensitive and accurate detection of copy number variants using read depth of coverage. Genome Res 19: 1586-1592.

25. Saeed AI, Sharov V, White J, Li J, Liang W, et al. (2003) TM4: a free, open-source system for microarray data management and analysis. Biotechniques 34: 374-378.

26. Storey JD, Tibshirani R (2003) Statistical significance for genomewide studies. Proc Natl Acad Sci U S A 100: 9440-9445.

27. Whisson SC, Boevink PC, Moleleki L, Avrova AO, Morales JG, et al. (2007) A translocation signal for delivery of oomycete effector proteins into host plant cells. Nature 450: 115-118.

28. Rasband W, National Institutes of Health U Image J: Image Processing and Analysis in Java.

29. Cooke LR, Carlisle DJ, Donaghy C, Quinn M, Perez FM, et al. (2006) The Northern Ireland *Phytophthora infestans* population 1998–2002 characterized by genotypic and phenotypic markers. Plant Pathology 55: 320-330.

30. Yang Z, Nielsen R (2000) Estimating synonymous and nonsynonymous substitution rates under realistic evolutionary models. Mol Biol Evol 17: 32-43.

31. Gilroy EM, Breen S, Whisson SC, Squires J, Hein I, et al. (2011) Presence/absence, differential expression and sequence polymorphisms between PiAVR2 and PiAVR2-like in *Phytophthora infestans* determine virulence on R2 plants. New Phytol 191: 763-776.

32. Avrova AO, Boevink PC, Young V, Grenville-Briggs LJ, van West P, et al. (2008) A novel *Phytophthora infestans* haustorium-specific membrane protein is required for infection of potato. Cell Microbiol 10: 2271-2284.

33. Raffaele S, Win J, Cano LM, Kamoun S (2010) Analyses of genome architecture and gene expression reveal novel candidate virulence factors in the secretome of *Phytophthora infestans*. BMC Genomics 11: 637.
